# Favorable strategies and barriers to the attainment of resilience in healthcare systems: scoping review

**DOI:** 10.1590/0102-311XEN227624

**Published:** 2025-10-03

**Authors:** Regina Glaucia Lucena Aguiar Ferreira, Hassã Pereira Lemos, Grayce Alencar Albuquerque, Andréa Sílvia Walter de Aguiar, Alice Maria Correia Pequeno, Neiva Francenely Cunha Vieira, Marcia C. Castro, Anya Pimentel Gomes Fernandes Vieira-Meyer

**Affiliations:** 1 Universidade Federal do Ceará, Fortaleza, Brasil.; 2 Secretaria Municipal de Saúde de Fortaleza, Fortaleza, Brasil.; 3 Centro Universitário Unichristus, Fortaleza, Brasil.; 4 Universidade Regional do Cariri, Juazeiro do Norte, Brasil.; 5 Centro Universitário da Grande Fortaleza, Fortaleza, Brasil.; 6 Harvard University, Boston, U.S.A.; 7 Fundação Oswaldo Cruz - Ceará, Eusébio, Brasil.

**Keywords:** Health System Resilience, Health Governance, Information Systems, Resiliencia de los Sistemas de la Salud, Gobernanza, Sistemas de Información

## Abstract

The resilience of a healthcare system regards the ability of health actors, institutions, and the population to maintain their essential functions in the face of adversity and reorganize based on the lessons learned. Resilient systems can achieve and maintain equity in the health and well-being of populations and respond to public health emergencies. The present scoping review involved a search of the PubMed, Virtual Health Library, Web of Science, and SciELO databases and employed the protocol of the Joanna Briggs Institute to answer the following research question: “What does the scientific evidence indicate as strategies and barriers to the attainment of a resilient healthcare system?”. The results revealed that the most strongly indicated strategies were decentralization of the system, a committed, motivated workforce, good governance and leadership, multisectoral partnerships, community involvement, an adequate information system, and investments that favor the sustainability of the healthcare system. In contrast, ineffective management without leadership, inadequate monitoring, an ineffective workforce, a lack of global solidarity, and failure to learn from past experiences constitute barriers to resilience. Understanding strategies and barriers is fundamental to the creation of a resilient healthcare system capable of dealing with chronic and acute stressors.

## Abstract (es)

La resiliencia de un sistema de salud se refiere a la capacidad de los actores de la salud, de las instituciones y de la población para mantener sus funciones esenciales ante una situación de adversidad y para reorganizarse en función de las lecciones aprendidas. Los sistemas resilientes pueden lograr y mantener la equidad en la salud y en el bienestar de las poblaciones y responder a las emergencias de salud pública. Esta revisión de alcance utilizó las plataformas PubMed y Biblioteca Virtual en Salud y las bases de datos Web of Science y SciELO, además del protocolo del Instituto Joanna Briggs para responder a la pregunta: “¿Qué señala la evidencia científica como estrategias y barreras para lograr un sistema de salud resiliente?”. Los resultados muestran que las estrategias más abordadas fueron la descentralización del sistema, la fuerza laboral comprometida y motivada, la gobernanza y liderazgo, las alianzas multisectoriales, la comunidad involucrada, el sistema de información adecuado y las inversiones que favorezcan la sostenibilidad del sistema de salud. Sin embargo, la gestión ineficaz y sin líderes, un inadecuado monitoreo, una ineficaz fuerza laboral, la falta de solidaridad global y la falta de aprendizaje de las experiencias previas fueron las barreras para la resiliencia. La comprensión de las estrategias y las barreras es fundamental para construir un sistema de salud resiliente capaz de hacer frente a los factores estresantes crónicos y agudos.

## Introduction

Healthcare systems are made up of interrelated sectoral or intersectoral components that determine and transform the health status of populations and include the population, service-providing institutions, and other actors, such as universities, professional councils, and the pharmaceutical industry ^1^. Such systems are subject to stressors that can disrupt their dynamics, exacerbating existing vulnerabilities and producing new weaknesses both for the system itself and the living conditions of the population ^2^. The impact of these events can emerge suddenly, such as an epidemic or a natural disaster, or slowly, such as the epidemiological transition some countries face. There are also chronic stressors, such as shortages of medications and healthcare providers ^3^.

The resilience of a healthcare system regards the ability of healthcare providers, institutions, and the population to maintain their essential functions when adversity arises and to reorganize ^4^ ^,^ ^5^. Thus, strengthening the system based on the lessons learned during a crisis is fundamental. Studies are needed to analyze barriers and determine strategies that favor resilience, enabling the continuous development of resilient systems responsive that are to the needs of the community.

The resilience of a system also depends on the ability to deal with events of different intensities and frequencies and not only major disasters ^6^. Smaller but recurring events can destabilize essential public health functions, compromise the continuity of services, and make systems prone to interruptions ^3^ ^,^ ^4^ ^,^ ^7^. Thus, developing resilience in public health must include proactive strategies that address and mitigate the impacts of daily practice ^6^.

Discussions on resilience resurfaced with the COVID-19 pandemic ^2^ ^,^ ^8^ ^,^ ^9^ ^,^ ^10^. A strong healthcare system that promotes resilience is imperative for governments. Resilient systems are fundamental to improving, achieving, and maintaining equity in the health and well-being of populations, as well as responding to public health emergencies, thus enabling sustainable socioeconomic development ^10^ ^,^ ^11^.

In 2015, the World Health Organization (WHO) proposed a basic set of six dimensions, denominated “building blocks”, on which the resilience of healthcare systems should be built: leadership and governance, health workforce, health information system, access to essential medicines and technologies, service delivery, and financing ^12^.

Other structures have been developed, such as the framework proposed by Kruk et al. ^4^, which encompasses five elements: awareness, integration, diversity, self-regulation, and adaptability. According the authors, the resilience of a healthcare system is characterized by the ability to anticipate and respond to crises. In this framework, resilience emerges in several ways: awareness of one’s own resources and potential threats enables strategic planning; integration with other sectors, such as transportation and education, enhances the response capacity; a diversity of approaches ensures flexibility to face different challenges; self-regulation enables the identification and minimization of interruptions to essential services; and adaptability enables the transformation of the system to meet new demands ^3^.

Using quantitative data from public databases and qualitative data from technical reports by Brazilian health authorities, Jatobá et al. ^13^ developed their framework based on Fuzzy logic (a mathematical model) to determine the score in four resilience capabilities (monitoring, anticipation, learning, and response), as well as an aggregate coefficient of the potential of resilience in health care.

Irrespective of the structure proposed in different frameworks, research on resilience provides a theoretical perspective for understanding healthcare systems as complex adaptive structures and highlights how health practices need to respond and adapt to stress, challenges, and demands according to their capabilities ^13^. Moreover, the literature describes factors that can facilitate or hinder the resilience of healthcare systems ^14^ ^,^ ^15^ ^,^ ^16^ ^,^ ^17^, which need to be better understood ^17^ ^,^ ^18^ ^,^ ^19^.

Therefore, the aim of the present study was to list favorable strategies and barriers to the resilience of healthcare systems and describe the mechanisms by which these factors either facilitate or hinder the attainment of resilience.

## Methods

The present scoping review was conducted in July 2023 using the PubMed and Virtual Health Library (VHL) platforms as well as the Web of Science and SciELO databases, following the Joanna Briggs Institute (JBI) protocol ^20^, which involves five steps: (1) identification and justification of the research question; (2) strategies for the identification of relevant studies; (3) eligibility and exclusion criteria; (4) selection and initial assessment of studies; and (5) data analysis.

### Identification and justification of the research question

The strategy considering Population, Concept, and Context (PCC) of the subject being researched was adopted. The following was the research question: “What does the scientific evidence indicate as strategies and barriers to the attainment of a resilient healthcare system?”.

The analysis of resilience seeks to understand the capacity of healthcare systems to prepare for absorbing, adapting to, learning from, transforming, and recovering from shocks. This analysis highlights elements such as leadership, decision-making, the coordination of actions as well as the availability and mobilization of resources, which are key elements for the management of a healthcare system ^21^.

Knowing the favorable strategies and barriers to the resilience of healthcare systems can contribute to discussions on the actions and policies necessary for a healthcare system to become more resilient through changes in financing, governance, the allocation of resources, the workforce, material and technological inputs, and the way healthcare services are provided.

### Strategies for the identification of relevant studies

The second stage consisted of the choice of databases and the development of the search strategies. As each database or platform has its own peculiarities, adaptations to the search strategies between the different databases or platforms are necessary for a successful search. Thus, the following descriptors and Boolean operators were used on the PubMed platform: *(Resilience OR Resilient) AND (“Health System” OR “Brazilian National Health System”)*. The descriptors *Resilience* and *“Health System”* interspersed by the Boolean operator *AND* were used in the Web of Science and SciELO databases as well as the VHL Regional Portal.

### Eligibility and exclusion criteria

Studies with information, reflections, and discussions regarding the characteristics, concepts, elements, attributes, strategies, and/or barriers to the attainment of a resilient healthcare system (Concept) in Brazil or another country (Context) involving healthcare providers, patients, or managers (Population) were considered. Only publications in Portuguese or English with the full text available were selected. The Rayyan systematic review management software (https://www.rayyan.ai/) was used to enable the selection and analysis of the studies retrieved from the databases.

For the extraction of data to be included in this review, the list of references originated in the Rayyan program was exported to a Microsoft Excel spreadsheet (https://products.office.com/), which was performed by two reviewers independently following a specific form developed for this stage to meet the objectives and answer the research question. The two researchers are professors of courses in the Health field (Nursing and Dentistry) at state and federal public universities with postgraduate degrees who work in the field of ​Public Health. Divergences of opinion regarding the selection of publications were resolved by a third reviewer (professor and researcher in Public Health at a federal research institution).

### Selection and initial assessment of studies

The following were the inclusion criteria: original articles, theoretical essays, literature reviews, scoping reviews, systematic reviews, dissertations, and theses available in full, with no restriction imposed regarding year of publication. Review studies were included to increase the number of publications, given the use of different databases in the different studies, which could expand the inclusion of publications on the subject. Editorials, abstracts, letters to the editor, government documents, undergraduate and specialization course completion papers, and articles not available in full online were excluded.

The titles and abstracts of all studies retrieved from the databases were analyzed. Documents that did not comply with the objective of the review were removed using the Rayyan software. In the first step, the titles and abstracts were screened for relevance. Publications whose abstracts listed or discussed aspects related to favorable strategies and/or barriers to the attainment of a resilient health system were selected for full-text analysis to determine compliance with the eligibility criteria. Publications that mentioned or discussed at least one favorable strategy or barrier to the resilience of health systems were included in the review, thus minimizing the risk of selection bias.

**Figure 1:**
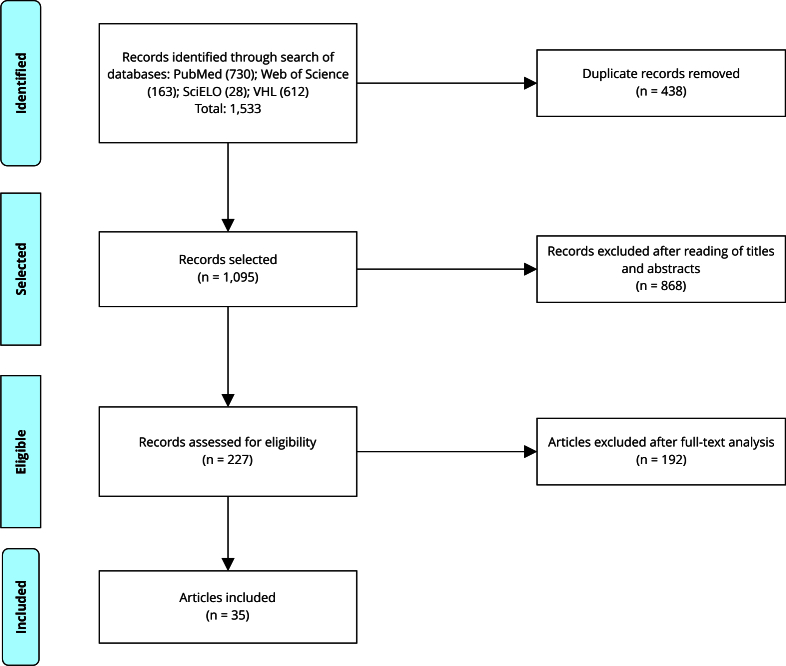
PRISMA diagram of article selection process.

Source: prepared by the authors, based on the recommendations from PRISMA - Extension for Scoping Reviews (PRISMA-ScR) ^22^.

The article selection process is displayed in the flowchart shown in Figure 1, with the quantitative results of each database, studies included/excluded, and the total selected for assessment and synthesis. This stage was developed following the *Preferred Reporting Items for Systematic Reviews and Meta-Analyses - Extension for Scoping Reviews* (PRISMA-ScR) ^22^. After compiling the information, the next stage consisted of the presentation of the synthesis of evidence and results in table format.

### Data analysis

Data analysis was performed using the characteristics outlined in the six building blocks of resilient healthcare systems recommended by the WHO ^12^. The protocol for this review was registered on the Open Science Framework platform (https://osf.io/jrkhb/files/osfstorage).

## Results

A total of 1,533 articles were retrieved from the databases: 730 from PubMed, 612 from VHL, 163 from Web of Science, and 28 from SciELO. As no time filter was used, publications from the mid-1990s to 2023 were found. After the removal of 438 duplicates, the titles and abstracts of 1,095 publications were analyzed, resulting in the exclusion of 868 that did not discuss the resilience of healthcare systems in the context of the object of this review. The remaining 227 publications were submitted to full-text analysis, 192 of which were excluded for not addressing the research question (did not mention or discuss strategies and/barriers that facilitate and/or hinder the resilience of healthcare systems). Thus, 35 articles composed the present scoping review. Box 1 displays data on the authorship, year of publication, title, and type/approach of the studies included.

**Box 1:** Publications included in scoping review - 2023 (n = 35).

STUDY (YEAR)	TITLE	STUDY APPROACH
Kruk & Freedman ^33^ (2008)	*Assessing Health System Performance in Developing Countries: A Review of the Literature*	Systematic review of the literature
Kruk et al. ^4^ (2015)	*What Is a Resilient Health System? Lessons from Ebola*	Theoretical essay
Ammar et al. ^30^ (2016)	*Health System Resilience: Lebanon and the Syrian Refugee Crisis*	Case study (literature review and quantitative analysis of secondary data)
Gilson et al. ^32^ (2017)	*Everyday Resilience in District Health Systems: Emerging Insights from the Front Lines in Kenya and South Africa*	Case study with qualitative approach (documental survey, in-depth interview, focus groups)
Abimbola et al. ^34^ (2019)	*The Impacts of Decentralization on Health System Equity, Efficiency and Resilience: A Realist Synthesis of the Evidence*	Literature review
Fridell et al. ^42^ (2020)	*Health System Resilience: What Are We Talking About? A Scoping Review Mapping Characteristics and Keywords*	Scoping review
Gilson et al. ^43^ (2020)	*Organizational Change and Everyday Health System Resilience: Lessons from Cape Town, South Africa*	Case study with qualitative approach (observation, interviews, and qualitative analysis of secondary data)
El Bcheraoui et al. ^31^ (2020)	*Assessing COVID-19 Through the Lens of Health Systems’ Preparedness: Time for a Change*	Theoretical essay
Jamal et al. ^57^ (2020)	*Health System Resilience in the Face of Crisis: Analysing the Challenges, Strategies and Capacities for UNRWA in Syria*	Case study with qualitative approach (interviews, focus groups, and documental analysis)
Thomas et al. ^38^ (2020)	*Strengthening Health Systems Resilience: Key Concepts and Strategies*	Theoretical study
Tumusiime et al. ^39^ (2020)	*Building Health System Resilience in the Context of Primary Health Care Revitalization for Attainment of UHC: Proceedings from the Fifth Health Sector Directors’ Policy and Planning Meeting for the WHO African Region*	Synthesis of discussion of planning and policy forum of African countries on resilient systems
Grimm et al. ^40^ (2021)	*Enhancing the Understanding of Resilience in Health Systems of Low- and Middle-Income Countries: A Qualitative Evidence Synthesis*	Literature review
Haldane et al. ^7^ (2021)	*Health Systems Resilience in Managing the COVID-19 Pandemic: Lessons from 28 Countries*	Literature review, government documents, and interviews with experts
Sundararaman et al. ^41^ (2021)	*Pandemic Resilience and Health Systems Preparedness: Lessons from COVID-19 for the Twenty-First Century*	Theoretical essay
Fleming et al. ^46^ (2022)	*Metrics and Indicators Used to Assess Health System Resilience in Response to Shocks to Health Systems in High Income Countries: A Systematic Review*	Systematic review
Saulnier et al. ^47^ (2022)	*‘We Have A Plan For That’: A Qualitative Study of Health System Resilience Through the Perspective of Health Workers Managing Antenatal and Childbirth Services During Floods in Cambodia*	Study with qualitative approach (semi-structured interviews)
Duby et al. ^48^ (2022)	*Adaptation and Resilience: Lessons Learned from Implementing a Combination Health and Education Intervention for Adolescent Girls and Young Women in South Africa During the COVID-19 Pandemic*	Study with qualitative approach (intervention and in-depth interviews)
Mustafa et al. ^16^ (2022)	*COVID-19 Preparedness and Response Plans from 106 Countries: A Review from a Health Systems Resilience Perspective*	Documental survey
Forsgren et al. ^15^ (2022)	*Health Systems Resilience in Practice: A Scoping Review to Identify Strategies for Building Resilience*	Scoping review
Burau et al. ^14^ (2022)	*Health System Resilience and Health Workforce Capacities: Comparing Health System Responses During the COVID‐19 Pandemic in Six European Countries*	Case study with qualitative approach (secondary and primary sources and information from experts)
Gooding et al. ^18^ (2022)	*How Can We Strengthen Partnership and Coordination for Health System Emergency Preparedness and Response? Findings from a Synthesis of Experience Across Countries Facing Shocks*	Documental survey
Amiri et al. ^49^ (2022)	*Health System Resilience in the Eastern Mediterranean Region: Perspective on the Recent Lessons Learned*	Round table
Foroughi et al. ^52^ (2022)	*Hospitals During Economic Crisis: A Systematic Review Based on Resilience System Capacities Framework*	Systematic review
Rawat et al. ^45^ (2022)	*The Contribution of Community Health Systems to Resilience: Case Study of the Response to the Drought in Ethiopia*	Case study with qualitative approach (interviews with key informants and discussions in focus groups)
Cannedy et al. ^58^ (2022)	*Health System Resiliency and the COVID-19 Pandemic: A Case Study of a New Nationwide Contingency Staffing Program*	Qualitative approach (interviews with directors and leaders)
Paschoalotto et al. ^10^ (2023)	*Health Systems Resilience: Is It Time to Revisit Resilience After COVID-19?*	Study with qualitative approach (semi-structured interviews with specialists in healthcare systems)
Sagan et al. ^50^ (2023)	*Assessing Resilience of a Health System Is Difficult But Necessary to Prepare for the Next Crisis*	Theoretical essay
Accoe et al. ^59^ (2023)	*Conditions for Health System Resilience in the Response to the COVID-19 Pandemic in Mauritania*	Case study with qualitative approach (semi-structures interviews with key informants of management teams)
Karreinen et al. ^60^ (2023)	*Living Through Uncertainty: A Qualitative Study on Leadership and Resilience in Primary Healthcare During COVID-19*	Case study with qualitative approach (semi-structured interviews with leaders of healthcare systems)
Ako-Egbe et al. ^56^ (2023)	*Liberia Health System’s Journey to Long-Term Recovery and Resilience Post-Ebola: A Case Study of an Exemplary Multi-Year Collaboration*	Case study (documental survey)
Mghamba et al. ^61^ (2023)	*The Use of Innovative Approaches to Strengthen Health System Resilience During the COVID-19 Pandemic: Case Studies from Selected Commonwealth Countries*	Case study with qualitative approach (interviews with epidemiologists and decision makers; documental survey)
Hellevik et al. ^62^ (2023)	*Multisectoral Action Towards Sustainable Development Goal 3.d and Building Health Systems Resilience During and Beyond COVID-19: Findings from an INTOSAI Development Initiative and World Health Organization Collaboration*	Study with qualitative approach (interviews com WHO auditing teams)
Lee et al. ^54^ (2023)	*Public Health Emergency Preparedness for Infectious Disease Emergencies: A Scoping Review of Recent Evidence*	Scoping review
Ezzati et al. ^17^ (2023)	*Resiliency of the Iranian Healthcare Facilities Against the COVID-19 Pandemic: Challenges and Solutions*	Study with qualitative approach (semi-structured interviews with 59 health policy makers, managers, and employees, and members of School of Medicine teaching staff)
Carvalho et al. ^19^ (2023)	*Transformative Dimensions of Resilience and Brittleness During Health Systems’ Collapse: A Case Study in Brazil Using the Functional Resonance Analysis Method*	Cross-sectional study (analysis of secondary data using Functional Resonance Analysis Method)

WHO: World Health Organization.

Based on the six building blocks of resilient healthcare systems proposed by the WHO, this scoping review identified factors considered facilitators (strategies) or hindrances (barriers) to the attainment of resilience. For each facilitating or hindering element listed (described as “central idea” in the tables of the results), the mechanisms by which these factors facilitate or hinder the resilience of the system were also reported.

Seven factors were identified as favorable strategies, four of which correspond to the “building blocks” of healthcare systems: workforce, governance, information systems, and financing. Decentralization, multisectoral partnerships, and community involvement also emerged as factors that promote resilience (Box 2).

**Box 2:** Strategies favorable to the attainment of the resilience of healthcare systems.

CENTRAL IDEA	SYNTHESIS OF EVIDENCE FROM THE LITERATURE	CONTRIBUTION MECHANISMS OF THE STRATEGY FOR THE ACHIEVEMENT OF RESILIENCE
1. Decentralization	Decentralization of decision-making is an important organizational capacity of resilience ^15^. Decentralized governments exhibit the necessary flexibility and timely responses required in the management of crises ^7^	Multiple governance centers in decentralized systems enable a “shock-absorbing” effect, such that weaknesses in one can be offset by other governance centers ^34^. Systems exhibited greater flexibility when decisions were made closer to the operational levels of the health system ^15^
2. Committed, motivated workforce	Health system resilience is enhanced by the capacity of the health workforce and strengthening the integration of the health professionals into governance ^14^. A committed, responsive workforce is a precondition for resilience ^30^ Even with scarce resources, the competence of the health workforce must be sufficiently high to maintain the day-to-day functions of the system ^42^ A committed, adequately sized, flexible, motivated, trained workforce is crucial to creating and maintaining resilience ^15^ ^,^ ^17^ ^,^ ^38^ ^,^ ^41^ ^,^ ^59^ ^,^ ^60^	The workforce is capable of absorbing the increased health needs generated by shocks, adapting to provide services with fewer resources than usual, and becoming transformed in accordance with changes in the environment ^45^ Developing the knowledge and skills of individuals and the entire system to deal with adverse conditions through training programs, practice, and experience leads to the improvement in the resilience of the system ^58^ One way to ensure an adequate workforce was the reallocation of staff across sectoral boundaries ^61^ Capacity for rapid mobilization and redistribution of health professionals and initiatives to sustain the productivity of health professionals even under stress are important mechanisms for strengthening resilience ^40^
3. Governance and leadership	Leadership and management are essential components and facilitators of resilience ^56^ ^,^ ^61^. Adaptation to emerging challenges requires strong, flexible leadership ^4^ Leaders are in a key position in the creation and maintenance of resilience before, during, and after a crisis ^61^. Participative leadership is more appropriate for complex organizations and enhances resilience ^17^ ^,^ ^43^ The experiences underscore the importance of leadership to day-to-day resilience exercised by managers of the healthcare system. Effective leadership must be able to demonstrate that the health system is capable of effectively preventing, detecting, or addressing a public health threat, with society the greatest beneficiary ^43^	Leadership based on respect for others or a sense of duty, enabling others to be decisive and innovative, creates a proper environment for managers and frontline health professionals, whose trust, commitment, and motivation are essential to resilient healthcare systems ^32^
4. Multisectoral partnerships	Strengthening health systems for resilience requires an integrated approach and multisectoral collaboration ^62^ For an effective response, coordinated multisectoral efforts are needed involving public, private, and civil society actors within and outside the health sector ^16^ ^,^ ^54^ ^,^ ^63^ The importance of national and international multisectoral collaboration, communication, and partnership is fundamental to building and sustaining the resilience of healthcare systems ^63^. Good governance based on a public-private partnership can be seen as a precondition for resilience ^30^	Collaboration among stakeholders, with meaningful engagement between the private sector, civil society, and service providers in formalized multi-stakeholder partnerships can help limit disruptions in access to healthcare services and enhance resilience of the system in the long-term ^52^ Good governance based on a public-private partnership helped ensure a constant flow of funds ^32^
5. Engaged community	There is only resilience in healthcare systems with the involvement of the community that knows all domains of the local context ^41^. Community health agents can increase the capacity of a system to respond to situations of crisis Health systems are staffed by health professionals and community members; the relationships and collaborations between them exert an influence on resilience ^49^ ^,^ ^60^	The involvement of communities enhances the resilience of the health system by providing critical information, ideas, and feedback to improve the capacity of the system to respond quickly to the needs of individuals during both shocks and calm times ^14^ ^,^ ^58^ Transparency, public trust, open communication, and legitimacy of the healthcare system are important for resilience. If the health system establishes appropriate, transparent relationships with people, the community will be more cooperative in times of crisis ^17^
6. Effective information system/monitoring and surveillance	A robust, digitized surveillance system that is perfectly integrated with health management information systems is an essential element for building resilience. It is important to use data to effectively anticipate, prepare for, and respond to changes, make evidence-based decisions, provide regular monitoring and evaluation, and conduct system analyses ^17^ ^,^ ^46^ Technology and information gain relevance when interacting with all dimensions of resilience in the healthcare system, improving its performance ^10^ Real-time health information is invaluable not only for enabling the early warning of an outbreak, but also for tracking the spread of the epidemic and preparing the healthcare system ^33^. Mobilizing monitoring systems, digital health detection, and expanding the capacity for the early identification of threats is mandatory for a resilient performance ^19^ Surveillance systems need to ensure that data reaches all interested parties and is quickly transformed into information for decision-making ^31^	An effective disease surveillance system reports not only unusual health events but also routine data on morbidity patterns. It identifies areas where access to other essential health services has been compromised and needs to be addressed urgently ^46^ A diligent information flow enables managers to react quickly in decision-making ^39^
7. Financing (investments in human, material, and financial resources - sustainability)	Resilience requires sufficient and available resources in situations of crisis ^17^ ^,^ ^39^ Human, financial, material, and technological resources to face a crisis are facilitating factors of the resilience of the system ^58^ ^,^ ^64^	In cases of crisis, having sufficient and well-allocated resources saves time for the development of capacities and provides resilience ^19^ A mechanism to mobilize resources quickly through reallocation and/or partner funds to respond to threats is an intervention that favors resilient systems ^40^ It is important to develop domestic manufacturing capacity for inputs and innovation ecosystems necessary for health security and self-sufficiency ^40^. Resilience of the health system requires the promotion of sustainability and investments to promote research and innovation ^62^

In contrast, five factors were identified as obstacles to the resilience of healthcare systems, three of which are exactly the opposite of the facilitating strategies described above and related to the “building blocks” recommended by WHO ^12^: ineffective leadership, workforce, and information systems. Two additional barriers also emerged: lack of global solidarity and failure to learn from past lessons. In the present review, barriers were less representative than strategies that favor the resilience of healthcare systems (Box 3).

**Box 3:** Barriers to the attainment of the resilience of healthcare systems.

CENTRAL IDEA	SYNTHESIS OF EVIDENCE FROM THE LITERATURE	MECHANISMS OF ACTION OF THE BARRIER TO HINDER THE ATTAINMENT OF RESILIENCE
1. Ineffective leadership/governance	Poor management and weak leadership, affecting the involvement of different sectors and levels of government, were identified as hindering coordination in response to stressors ^15^ ^,^ ^16^ The most important resilience challenges were a fragmented management system ^60^, person-centered decision-making, lack of coordination, ineffective leadership, and a lack of commitment on the part of managers ^15^	The lack of central guidelines hinders a resilient performance, as decision-making and policy-making become more taxing ^17^ A lack of intra- and inter-sectoral coordination is a major challenge for leadership, as it increases the likelihood of delays and wasted time on projects and programs. Self-centeredness and partisanship prevent managers from developing a national vision ^15^
2. Ineffective information/monitoring and evaluation system	Inadequate monitoring and evaluation constitute important challenges to resilience ^15^	The lack of data/information/communication and government transparency reduces effective decision-making and social capital and constitutes a major challenge to resilience ^15^
3. Ineffective workforce	The ineffective selection and recruitment of employees, the lack of awareness among employees, and their low competence and skill, unpreparedness, and lack of support are the main challenges ^15^	Employees are the most important resources of an organization; they must be developed and trained to achieve organizational objectives ^15^
4. Lack of global solidarity	The decrease in global solidarity may have affected the resilience of healthcare systems ^42^	The frailty of healthcare systems occurs at the same time in which uncertainties increase, caused by lower global solidarity and divergent views of world leaders ^42^
5. Failure to learn from past experiences (lessons); lack of culture of resilience	Failure to take advantage of experiences and lessons learned and lack of organizational learning constitute challenges to healthcare systems ^15^	If organizations do not understand resilience, they will not be able to implement it, will not understand its definitions and concepts, will suffer from entropy, and will become obsolete ^15^

## Discussion

Despite the different meanings given to the term health system resilience, this terminology was treated in the present review as an attribute of public healthcare systems. Resilience enables such systems to adapt, absorb, and respond to routine and extraordinary demands without compromising the provision of universal, equitable care ^23^. Resilience also enables systems to reorganize based on the lessons learned ^4^. With regards to crisis situations, public healthcare systems must be more adaptive, proactive, and responsive to ensure the universal, comprehensive, equitable coverage of essential services and avoid collapse ^24^.

Jatobá et al. ^23^ state that discussions on the resilience of healthcare systems persist even amidst uncertainty with regards to the meaning of the term and with no consensus on its operationalization in the context of public health emergencies. However, it is essential for resilience to be treated not only as a reactive concept, but also as a proactive one, especially when speaking of the capacity of healthcare systems to respond to unusual circumstances. Resilience should not be seen as resignation in the face of adversity through adaptive responses, but as a driver of the continuous development of monitoring, anticipation, and learning capabilities involved in the constant transformation of healthcare systems ^6^.

Most studies on resilience in public health focus on catastrophic events, such as pandemics and epidemics, rather than the continuous delivery of comprehensive health services ^25^. In this review, the notion of resilience in the functioning of healthcare systems was also primarily directed at public health emergencies, possibly justified by the health crisis experienced with the COVID-19 pandemic.

Strategies considered favorable to the attainment of the resilience of healthcare systems emerged in the studies analyzed in this scoping review, such as governance and financing. According to Haldane et al. ^7^, several countries have enhanced their resilience through governance, financing, and accountability mechanisms by implementing support packages for vulnerable groups, thus protecting and supporting low-income populations and reducing the burden on healthcare systems. Examples include specific health programs for indigenous peoples in countries such as Brazil ^26^ and Canada ^27^, differentiated policies for migrant and marginalized populations ^28^ and homeless people, and the expansion of community health agents programs in vulnerable areas in the United Kingdom ^29^.

Although often overlooked in the context of responses to a shocks ^30^, good governance, especially intersectoral governance, merits special attention ^31^. Well-managed healthcare systems are better prepared to face both long-term health challenges and sudden emergencies. As a key element in policymaking, effective public health governance is fundamental to ensuring adequate resource allocation and fostering intersectoral cooperation to address the social determinants of health, including the prioritization of factors such as social vulnerability and diversity ^6^.

Participative leadership is more appropriate for complex adaptive organizations and can enhance the resilience of such organizations ^17^. Kruk et al. ^4^ stress the importance of strong, flexible leadership to face emerging challenges, not only in times of crisis, but also under normal circumstances (daily resilience). Gilson et al. ^32^ state that leadership based on trust that prioritizes empowering, training, and motivating subordinates, especially those on the front line, makes them decisive and innovative, contributing to enhancing the resilience of the healthcare system.

The decentralization of decision-making, which is a strategy emphasized in this scoping review, constitutes an important organizational resilience capacity, as it provides the necessary flexibility for the demands that arise in the management of a crisis ^15^ ^,^ ^33^. Resilient organizations use decentralization, self-organization, and shared decision-making to enable flexibility and adaptation ^30^. Abimbola et al. ^34^ state that multiple governance centers in decentralized systems enable shock absorption, as the weaknesses of one governance center can be offset by the strengthened governance of other centers.

In the Brazilian case, exploring the roles, responsibilities, and actions of management boards among the different spheres of government in the occurrence of crisis situations is a fundamental topic for the research agenda on resilience in the Brazilian Unified National Health System (SUS, acronym in Portuguese). Particularly in situations of crisis, it is important to understand how management uses information and evidence for decision-making; how key actors participate in the system; and how the response is coordinated between the public and private sectors of the healthcare system ^35^.

According to Bloom et al. ^36^, resilience in public health is a contextual capacity largely dependent on the construction of national universal public healthcare systems. This requires continuous efforts to strengthen governmental structures and promote intersectoral partnerships. Thus, resilience is an essential feature of democratic governance that encompasses the development of skills as well as the response to threats and political pressures. The responses of healthcare systems cannot be isolated from public policies, changes, or the emergence of ruptures and conflicts ^37^.

In the present scoping review, ongoing multisectoral collaboration was identified as a factor that contributes to closing persistent gaps on all levels of the healthcare system ^16^. An effective response requires coordinated multisectoral efforts involving public, private, and civil society actors both within and outside the health sector ^38^. Formalized multisectoral partnerships can prevent disruptions in access to healthcare services, thus enhancing the resilience of the system in the long term ^39^, especially in times of crisis ^40^. Furthermore, recognizing that healthcare systems are embedded in other complex structures (e.g., political, economic, judicial, social, and ecological systems) and on different scales (local, subnational, national, and international levels) facilitates the understanding of how healthcare systems are affected by factors that are directly related to public health, although they may not seem to be ^7^ ^,^ ^41^.

The attainment of resilience as a capacity of the healthcare system is not a simple process. It requires coordination between material and human resources and the allocation of tasks in accordance with the nature of the threat. Specific actors in the health system deal with different types of events, interacting with patients as well as public and private administrators. Such interactions depend on the motivations and interests of individuals as well as the structures within which health systems operate and stable, open, adaptive financial, and political arrangements to improve the performance of the system ^23^.

According to Jatobá & Carvalho ^25^, the continuous development of adaptive, absorbent, and preventive capacities enables healthcare providers to deal with unknown situations proactively, moving from traditional approaches to more resilient behaviors. However, systems that depend only on the resilience of the actors, especially those working on the front lines, overburden workers, leading to instability and the loss of control ^37^. Stable, responsive, adaptive, high-level (financial and political) arrangements need to promote, through learning, transformations that lead to the good performance of the system ^23^.

A committed/motivated workforce is considered an important facilitating element of resilience, as it contributes to absorbing the growing health needs generated by shocks, adapting in order to provide services with resources that are often smaller than usual, and transforming in accordance with changes in the environment generated by the shock ^34^. The flexibilization of tasks and teamwork are also fundamental to achieving resilience ^42^. Moreover, preparing individuals to deal with adverse conditions through training programs can contribute to improving the resilience of the system ^43^.

Resilience-centered governance in public health emphasizes social participation in policymaking processes, ensuring that those affected by social determinants also participate in the planning of policies and programs that impact their lives ^6^. A resilient performance depends on public policies based on a comprehensive understanding of the local situation of communities ^35^ ^,^ ^44^. Therefore, good governance is essential to addressing structural factors that exert an influence on health outcomes, especially in disadvantaged populations. By focusing on equity and health outcomes, governance can drive systemic changes that help diminish health disparities ^6^.

Community involvement is an important strategy for achieving resilience in health systems. According to Rawat et al. ^45^, effectively engaged, proactive communities linked to their healthcare systems are essential to the construction of resilience and a successful response. Trust and a sense of belonging to the community can be built through the involvement of community members in policy formulation processes and the management of healthcare services ^46^ ^,^ ^47^.

Information systems constitute is a strategy addressed by several authors in this scoping review ^17^ ^,^ ^48^ ^,^ ^49^ and emphasized by Kruk & Freedman ^33^. Such systems not only provide early warnings of an outbreak, but can also track the spread of epidemics, predicting and preparing the healthcare system for the outbreak ^50^. Fukuma et al. ^51^ draw attention to the fact that, during the great Fukushima earthquake in Japan, advances in information technology played a fundamental role in the local community as an alternative source of information and a communication platform. However, it is necessary to establish a clear flow of information to avoid the risk of wrong decisions and incomplete or duplicate records, ensuring that information is shared appropriately and in a timely manner to enable a fast, proper response ^52^. Carvalho et al. ^19^ state that the COVID-19 pandemic exposed the frailty of healthcare systems on the national and subnational levels, Therefore, mobilizing monitoring systems and expanding the capacity for early identification of trans-border threats have become mandatory for a resilient performance of the healthcare system ^52^.

According to Jatobá ^6^, institutional capacity alone is not sufficient to achieve a resilient performance in public health. Indeed, recent experiences have shown that strong institutional capacity does not ensure resilient behavior ^25^ ^,^ ^53^. During the COVID-19 pandemic, some developed countries with considerable institutional capacity struggled to control the rapid spread of the SARS-CoV-2 virus. Resilience depends on how healthcare systems perform in situations of adversity, managing their institutional capacities for the sake of the continuity of essential public health functions ^3^ ^,^ ^44^. This was evident in countries such as the United States and Brazil during the pandemic, which, despite having resources, faced operational difficulties due to conflicting strategies at higher decision-making levels ^19^.

In an analysis of the Brazilian case, Massuda et al. ^21^ point to austerity policies as elements that weakened the response of the SUS to the pandemic, despite the broad, comprehensive structure constructed over the years. The authors stress the current situation resulting from the disastrous role of the government, but also chronic problems of the SUS, such as underfunding, insufficient staff, etc.

In the present study, investments in material and human resources emerged as factors that promote the construction of resilient health systems, mitigating the impacts of a health emergency and reducing the disruption of essential public health services ^54^. However, the literature points out the need for investments in domestic manufacturing capacity and the creation of ecosystems of innovation necessary for health security and self-sufficiency ^50^.

In line with this argument, Nyasulu et al. ^55^ point to the infrastructure of the healthcare systems of countries as a determinant in the response to pandemics, irrespective of available resources. The authors cite the example of Malawi; despite having a healthcare system that lacked resources, the existing community health structures contributed to the resilience of healthcare systems during the COVID-19 pandemic.

According to Paschoalotto et al. ^35^, it is important to explore how public and private financial resources in Brazil are allocated in the face of a crisis situation and how financing inequalities influence the resilience of the SUS. It is also important to examine the flow of financial resources among federative spheres in response to a crisis situation and the criteria used to allocate resources.

Regarding the barriers to the resilience of healthcare systems, low worker competence and skills, combined with a lack of support for these workers, were the main challenges described by Ezzati et al. ^17^. The authors also stress the importance of a “culture of resilience”, which values ​​training and changes in behavior for the sake of the resilience of the healthcare system.

Ineffective management without good leadership was a major challenge during the COVID-19 pandemic ^17^. Fragmented, isolated approaches hampered efforts to make healthcare systems more resilient ^56^ and a lack of solidarity led to cracks in the conjuncture between global and national health care and disparities between local and global responses to the pandemic ^31^. Moreover, failure to build upon experiences and the lessons learned stood out as a barrier to the resilience of healthcare systems ^17^.

Fridell et al. ^42^ point out that most studies addressing resilience focus on absorbing systemic shocks in the relatively short term rather than on learning and the transformation of the system in the long term. To reverse this situation, greater attention must be given to factors that favor the ability to learn from shocks so that the system can respond effectively in the future ^39^.

Guinea is an example of a country that used its experience based on lessons learned to contain the negative impacts of COVID-19. After the shock caused by the Ebola epidemic (2014-2016), a plan was put in place to train local epidemiologists. According to Tumusiime et al. ^39^, there were no reports of challenges or difficulties related to infrastructure, equipment, or other resources. Clearly, having these structures in place before COVID-19 provided several benefits, such as reduced expenses. However, additional lessons were learned with COVID-19, such as the awareness of the need to have mortality and morbidity data available in real-time ^39^.

According to Jatobá et al. ^13^, achieving ambitious health goals requires strong, functional, inclusive healthcare systems. The building blocks of resilient health systems proposed by the WHO highlight only the essential elements. Further efforts and investments are needed to ensure universal access and coverage. However, these building blocks constitute a useful starting point for health administrators and authorities.

Despite the relevance of the subject of this scoping review, which followed the initially proposed protocol, the study has some limitations, such as having restricted the languages ​​to Portuguese and English and not having used all available databases/platforms or the gray literature. Another barrier was the extensive variability in the concept of the resilience, combined with the strong tendency to use it in the context of crises, given the recent COVID-19 pandemic, causing the peak of publications to be concentrated in the years 2020 to 2022. Despite these aspects, this study achieved its objective, enabling a careful compilation of publications on the subject, thus contributing to the understanding of strategies and barriers to the attainment of resilience in healthcare systems, which is necessary to face future shocks/stressors in the Health field.

## Final considerations

The decentralization of the system, a committed, motivated workforce, effective governance and leadership, multisectoral partnerships, an involved community, an adequate information system, and investments that favor the sustainability of the healthcare system are strategies that contribute to the attainment of resilience. In contrast, ineffective management/lack of leadership, inadequate monitoring, an ineffective workforce, a lack of global solidarity, and failure to learn from past experiences constitute barriers to the resilience of healthcare systems.

Understanding the components of a resilient healthcare system and how to build one while maintaining the provision of essential services is crucial to the preparation of healthcare systems in the occurrence of adversities. Thus, an understanding on the part of all stakeholders with regards to strategies and barriers is fundamental to building a more resilient and effective healthcare system capable of facing chronic and acute stressors, thus enabling the population to benefit from increasingly qualified healthcare services.

## References

[1] Bispo JP, Messias KLM (2005). Sistemas de serviços de saúde: principais tipologias e suas relações com o sistema de saúde brasileiro.. Revista Saúde.com.

[2] Bispo JP (2022). Resiliência do Sistema Único de Saúde no contexto da pandemia de COVID-19: como se fortalecer?. Cad Saúde Pública.

[3] Kruk ME, Ling EJ, Bitton A, Cammett M, Cavanaugh K, Chopra M (2017). Building resilient health systems a proposal for a resilience index. BMJ.

[4] Kruk ME, Myers M, Varpilah ST, Dahn BT (2015). What is a resilient health system Lessons from Ebola. Lancet.

[5] Kruk ME, Gage AD, Arsenault C, Varpilah ST, Dahn BY (2018). High-quality health systems in the Sustainable Development Goals era time for a revolution. Lancet Glob Health.

[6] Jatobá A (2025). Resilience in public health what is and what should never be. Health Policy Technol.

[7] Haldane V, De Foo C, Abdalla SM, Jung AS, Tan M, Wu S (2021). Health systems resilience in managing the COVID-19 pandemic lessons from 28 countries. Nat Med.

[8] Barasa EW, Cloete K, Gilson L (2017). From bouncing back to nurturing emergence reframing the concept of resilience in health systems strengthening. Health Policy Plan.

[9] Bigoni A, Malik AM, Tasca R, Carrera MBM, Schiesari LMC, Gambardell DD (2022). Brazil's health system functionality amidst the COVID-19 pandemic an analysis of resilience. Lancet Reg Health Am.

[10] Paschoalotto MAC, Lazzari EA, Rocha R, Massuda A, Castro MC (2023). Health systems resilience is it time to revisit resilience after COVID-19?. Soc Sci Med.

[11] Gouya MM, Seif-Farahi K, Hemmati P (2023). An overview of Iran's actions in response to the COVID-19 pandemic and in building health system resilience. Front Public Health.

[12] World Health Organization (2015). Operational framework for building climate resilient health systems.

[13] Jatobá A, de Castro Nunes P, de Carvalho PVR (2023). A framework to assess potential health system resilience using fuzzy logic.. Rev Panam Salud Pública.

[14] Burau V, Falkenbach M, Neri S, Peckham S, Wallenburg I, Kuhlmann E (2022). Health system resilience and health workforce capacities comparing health system responses during the COVID-19 pandemic in six European countries. Int J Health Plann Manage.

[15] Forsgren L, Tediosi F, Blanchet K, Saulnier DD (2022). Health systems resilience in practice a scoping review to identify strategies for building resilience. BMC Health Serv Res.

[16] Mustafa S, Zhang Y, Zibwowa Z, Seifeldin R, Ako-Egbe L, McDarby G (2022). COVID-19 preparedness and response plans from 106 countries a review from a health systems resilience perspective. Health Policy Plan.

[17] Ezzati F, Mosadeghrad AM, Jaafaripooyan E (2023). Resiliency of the Iranian healthcare facilities against the COVID-19 pandemic challenges and solutions. BMC Health Serv Res.

[18] Gooding K, Bertone MP, Loffreda G, Witter S (2022). How can we strengthen partnership and coordination for health system emergency preparedness and response Findings from a synthesis of experience across countries facing shocks. BMC Health Serv Res.

[19] Carvalho PVR, Bellas H, Viana J, de Castro Nunes P, Arcuri R, da Silva Fonseca V (2023). Transformative dimensions of resilience and brittleness during health systems' collapse: a case study in Brazil using the Functional Resonance Analysis Method.. BMC Health Serv Res.

[20] Aromataris E, Munn Z JBI Manual for Evidence Synthesis..

[21] Massuda A, Malik AM, Vecina-Neto G, Tasca R, Ferreira-Junior WC (2021). A resiliência do Sistema Único de Saúde frente à COVID-19.. Cadernos EBAPE.BR.

[22] Tricco AC, Lillie E, Zarin W, O'Brien KK, Colquhoun H, Levac D (2018). PRISMA Extension for Scoping Reviews (PRISMA-ScR): checklist and explanation.. Ann Intern Med.

[23] Jatobá A, Castro-Nunes P, Rodrigues de Carvalho PV (2025). On the epistemology of resilience in public health: a novel perspective in a changing world.. Front Health Serv.

[24] Nohrstedt D (2013). Does adaptive capacity influence service delivery Evidence from Swedish emergency management collaborations. Public Manag Rev.

[25] Jatobá A, Carvalho PVR (2024). The resilience of the Brazilian Unified Health System is not responding to disasters. Rev Saúde Pública.

[26] Pontes ADM, Santos RV (2020). Health reform and indigenous health policy in Brazil: contexts, actors and discourses.. Health Policy Plan.

[27] Burnett K, Sanders C, Halperin S (2020). Indigenous peoples, settler colonialism, and access to health care in rural and northern Ontario. Health Place.

[28] Abubakar I, Gram I, Iasoye S, Achiume ET, Becares I, Bola GK (2022). Confronting the consequences of racism, xenophobia, and discrimination on health and health-care systems. Lancet.

[29] Junghans C, Antonacci G, Williams A, Harris M (2023). Learning from the universal, proactive outreach of the Brazilian community health worker model impact of a community health and wellbeing worker initiative on vaccination, cancer screening and NHS health check uptake in a deprived community in the UK. BMC Health Serv Res.

[30] Ammar W, Kdouh O, Hammoud R, Hamadeh R, Harb H, Ammar Z (2016). Health system resilience Lebanon and the Syrian refugee crisis. J Glob Health.

[31] El Bcheraoui C, Weishaar H, Pozo-Martin F, Hanefeld J (2020). Assessing COVID-19 through the lens of health systems' preparedness time for a change. Global Health.

[32] Gilson L, Barasa E, Nxumalo N, Cleary S, Goudge J, Molyneux S (2017). Everyday resilience in district health systems emerging insights from the front lines in Kenya and South Africa. BMJ Glob Health.

[33] Kruk ME, Freedman LP (2008). Assessing health system performance in developing countries a review of the literature. Health Policy.

[34] Abimbola S, Baatiema L, Bigdeli M (2019). The impacts of decentralization on health system equity, efficiency and resilience a realist synthesis of the evidence. Health Policy Plan.

[35] Paschoalotto MAC, Lazzari EA, Castro MC, Rocha R, Massuda A (2022). The health systems resilience notes for a research agenda for the SUS. Saúde Debate.

[36] Bloom G, MacGregor H, McKenzie A, Sokpo E Strengthening health systems for resilience..

[37] Carvalho PVR (2011). The use of Functional Resonance Analysis Method (FRAM) in a mid-air collision to understand some characteristics of the air traffic anagement system resilience. Reliab Eng Syst Saf.

[38] Thomas S, Sagan A, Larkin J, Cylus J, Figueras J, Karanikolos M (2020). Strengthening health systems resilience: key concepts and strategies.

[39] Tumusiime P, Karamagi H, Titi-Ofei R, Amri M, Seydi ABW, Kipruto H (2020). Building health system resilience in the context of primary health care revitalization for attainment of UHC proceedings from the Fifth Health Sector Directors' Policy and Planning Meeting for the WHO African Region. BMC Proc.

[40] Grimm PY, Oliver S, Merten S, Han WW, Wyss K (2021). Enhancing the understanding of resilience in health systems of low- and middle-income countries a qualitative evidence synthesis. Int J Health Policy Manag.

[41] Sundararaman T, Muraleedharan VR, Ranjan A (2021). Pandemic resilience and health systems preparedness lessons from COVID-19 for the twenty-first century. J Soc Econ Dev.

[42] Fridell M, Edwin S, von Schreeb J, Saulnier DD (2020). Health system resilience what are we talking about? A scoping review mapping characteristics and keywords. Int J Health Policy Manag.

[43] Gilson L, Ellokor S, Lehmann U, Brady L (2020). Organizational change and everyday health system resilience lessons from Cape Town, South Africa. Soc Sci Med.

[44] Haldane V, Morgan GT (2021). From resilient to transilient health systems the deep transformation of health systems in response to the COVID-19 pandemic. Health Policy Plan.

[45] Rawat A, Karlstrom J, Ameha A, Oulare M, Omer MD, Desta HH (2022). The contribution of community health systems to resilience case study of the response to the drought in Ethiopia. J Glob Health.

[46] Fleming P, O'Donoghue C, Almirall-Sanchez A, Mockler D, Keegan C, Cylus J (2022). Metrics and indicators used to assess health system resilience in response to shocks to health systems in high-income countries: a systematic review.. Health Policy.

[47] Saulnier DD, Thol D, Por I, Hanson C, von Schreeb J, Alvesson HM (2022). 'We have a plan for that': a qualitative study of health system resilience through the perspective of health workers managing antenatal and childbirth services during floods in Cambodia.. BMJ Open.

[48] Duby Z, Bunce B, Fowler C, Jonas K, Govindasamy D, Wagner C (2022). Adaptation and resilience lessons learned from implementing a combination health and education intervention for adolescent girls and young women in South Africa during the COVID-19 pandemic. Front Health Serv.

[49] Amiri M, Al Nsour M, Alonso-Garbayo A, Al Serouri A, Maiteh A, Badr E (2022). Health system resilience in the Eastern Mediterranean Region perspective on the recent lessons learned. Interact J Med Res.

[50] Sagan A, Thomas S, Webb E, McKee M (2023). Assessing resilience of a health system is difficult but necessary to prepare for the next crisis. BMJ.

[51] Fukuma S, Ahmed S, Goto R, Inui TS, Atun R, Fukuhara S (2017). Fukushima after the Great East Japan Earthquake lessons for developing responsive and resilient health systems. J Glob Health.

[52] Foroughi Z, Ebrahimi P, Aryankhesal A, Maleki M, Yazdani S (2022). Hospitals during economic crisis a systematic review based on resilience system capacities framework. BMC Health Serv Res.

[53] Saulnier DD, Topp SM (2024). We need to talk about bad resilience. BMJ Glob Health.

[54] Lee JM, Jansen R, Sanderson KE, Guerra F, Keller-Olaman S, Murti M (2023). Public health emergency preparedness for infectious disease emergencies a scoping review of recent evidence. BMC Public Health.

[55] Nyasulu JCY, Chirwa J, Kumwenda J, Chikalipo M (2022). Resilience of health systems during the public health emergency of the COVID-19 pandemic the role of existing community health structures in rural Malawi. Am J Disaster Med.

[56] Ako-Egbe L, Seifeldin R, Saikat S, Wesseh SC, Bolongei MB, Ngormbu BJ (2023). Liberia health system's journey to long-term recovery and resilience post-Ebola a case study of an exemplary multi-year collaboration. Front Public Health.

[57] Jamal Z, Alameddine M, Diaconu K, Lough G, Witter S, Ager A (2020). Health system resilience in the face of crisis analysing the challenges, strategies and capacities for UNRWA in Syria. Health Policy Plan.

[58] Cannedy S, Bergman A, Medich M, Rose DE, Stockdale SE (2022). Health system resiliency and the COVID-19 pandemic a case study of a new nationwide contingency staffing program. Healthcare (Basel).

[59] Accoe K, Criel B, Ag Ahmed MA, Buitrago VT, Marchal B (2023). Conditions for health system resilience in the response to the COVID-19 pandemic in Mauritania. BMJ Glob Health.

[60] Karreinen S, Paananen H, Kihlström L, Janhonen K, Huhtakangas M, Viita-Aho M (2023). Living through uncertainty a qualitative study on leadership and resilience in primary healthcare during COVID-19. BMC Health Serv Res.

[61] Mghamba J, Gilmour E, Robinson L, Simba A, Tuyishime A, Persaud A (2023). The use of innovative approaches to strengthen health system resilience during the COVID-19 pandemic case studies from selected Commonwealth countries. Front Public Health.

[62] Hellevik S, Mustafa S, Zhang Y, Shirsat A, Saikat S (2023). Multisectoral action towards sustainable development goal 3 d and building health systems resilience during and beyond COVID-19: findings from an INTOSAI development initiative and World Health Organization collaboration. Front Public Health.

[63] Hanefeld J, Mayhew S, Legido-Quigley H, Martineau F, Karanikolos M, Blanchet K (2018). Towards an understanding of resilience responding to health systems shocks. Health Policy Plan.

[64] Thomas S, Keegan C, Barry S, Layte R, Jowett M, Normand C (2013). A framework for assessing health system resilience in an economic crisis Ireland as a test case. BMC Health Serv Res.

