# Supplementary material for: Favorable strategies and barriers to the attainment of resilience in healthcare systems: scoping review
Source: Cad Saude Publica. 2025 Oct 3;41(9):e00227624. doi: 10.1590/0102-311XEN227624 (PMC12494389; doi:10.1590/0102-311XEN227624)
Supplement: Translation

# Translation: Estratégias favoráveis e barreiras para o alcance da resiliência de sistemas de saúde: revisão de escopo

**DOI:** 10.1590/0102-311XPT227624

**Authors:** Regina Glaucia Lucena Aguiar Ferreira, Hassã Pereira Lemos, Grayce Alencar Albuquerque, Andréa Sílvia Walter de Aguiar, Alice Maria Correia Pequeno, Neiva Francenely Cunha Vieira, Marcia C. Castro, Anya Pimentel Gomes Fernandes Vieira-Meyer

**Affiliations:** 1 Universidade Federal do Ceará, Fortaleza, Brasil.; 2 Secretaria Municipal de Saúde de Fortaleza, Fortaleza, Brasil.; 3 Centro Universitário Unichristus, Fortaleza, Brasil.; 4 Universidade Regional do Cariri, Juazeiro do Norte, Brasil.; 5 Centro Universitário da Grande Fortaleza, Fortaleza, Brasil.; 6 Harvard University, Boston, U.S.A.; 7 Fundação Oswaldo Cruz - Ceará, Eusébio, Brasil.

**Keywords:** Resiliência de Sistemas de Saúde, Governança em Saúde, Sistemas de Informação

## Abstract

A resiliência de um sistema de saúde refere-se à capacidade dos atores de saúde, das instituições e da população de manter suas funções essenciais quando uma adversidade surge e de se reorganizar, embasados pelas lições aprendidas. Sistemas resilientes podem alcançar e manter a equidade na saúde e bem-estar das populações e responder a emergências de saúde pública. Esta revisão de escopo utilizou as plataformas PubMed e Biblioteca Virtual em Saúde e as bases de dados Web of Science e SciELO, além do protocolo do Instituto Joanna Briggs para responder à questão: “O que as evidências científicas apontam como estratégias e barreiras para o alcance de um sistema de saúde resiliente?”. Constatou-se que as estratégias mais fortemente abordadas são: descentralização do sistema, força de trabalho comprometida e motivada, governança e liderança, parcerias multissetoriais, comunidade envolvida, sistema de informação adequado e investimentos que favoreçam a sustentabilidade do sistema de saúde. Contudo, gestão ineficaz e sem liderança, monitoramento inadequado, força de trabalho ineficaz, falta de solidariedade global e falha em aprender com as experiências passadas são barreiras à resiliência. Compreender as estratégias e barreiras é fundamental para a construção de um sistema de saúde resiliente, capaz de enfrentar estressores crônicos e agudos.

## Introdução

Sistemas de saúde são conjuntos de componentes inter-relacionados, setoriais ou intersetoriais, que determinam e transformam a situação de saúde das populações. Deles fazem parte a população e as instituições prestadoras de serviços, além de outros atores, como: as universidades, os conselhos profissionais e a indústria farmacêutica ^1^. Tais sistemas estão sujeitos a fatores estressores capazes de desajustar sua dinâmica, ampliando as vulnerabilidades existentes e produzindo novas fragilidades, tanto para os próprios sistemas, como para as condições de vida da população ^2^. O impacto desses eventos pode surgir de forma súbita, como uma epidemia ou um desastre natural, ou lenta, como a transição epidemiológica enfrentada por alguns países. Existem, ainda, os estressores crônicos, como a escassez de medicamentos ou de profissionais de saúde ^3^.

A resiliência de um sistema de saúde é a capacidade dos atores de saúde, das instituições e da população de manter suas funções essenciais quando uma adversidade surge e de se reorganizar, embasados pelas lições aprendidas durante essa crise ^4^ ^,^ ^5^. Nesse sentido, fortalecer um sistema de saúde com base nas lições aprendidas durante as crises é fundamental, sendo essenciais estudos que analisem as barreiras e estratégias favoráveis à resiliência, possibilitando o contínuo desenvolvimento de sistemas resilientes e responsivos às necessidades da comunidade.

É importante ressaltar que o comportamento resiliente do sistema depende também da capacidade de lidar com eventos de diferentes intensidades e frequências, e não apenas grandes desastres ^6^. Eventos menores, mas recorrentes, podem desestabilizar funções essenciais de saúde pública, comprometer a continuidade da prestação de serviços e tornar os sistemas propensos à interrupção ^3^ ^,^ ^4^ ^,^ ^7^. Assim, desenvolver resiliência em saúde pública deve também incluir estratégias proativas que abordem e mitiguem os impactos do cotidiano ^6^.

O debate em torno da resiliência ressurgiu com a pandemia de COVID-19 ^2^ ^,^ ^8^ ^,^ ^9^ ^,^ ^10^. Sabe-se que o sistema de saúde fortalecido e promotor da resiliência é imperativo para os governos, pois sistemas resilientes são fundamentais para melhorar, alcançar e manter a equidade na saúde e bem-estar das populações, assim como responder a emergências de saúde pública, permitindo o desenvolvimento socioeconômico sustentável ^10^ ^,^ ^11^.

Nesse contexto, a Organização Mundial da Saúde (OMS) propôs em 2015 um conjunto básico de seis dimensões pelas quais a resiliência dos sistemas de saúde deve se desenvolver, denominadas de “blocos de construção”. São eles: liderança e governança; força de trabalho em saúde; sistema de informação em saúde; produtos e tecnologias médicas essenciais; prestação de serviços; e financiamento ^12^.

Outras estruturas foram elaboradas, a exemplo do *framework* de Kruk et al. ^4^, que engloba cinco elementos: consciência, integração, diversidade, autorregulação e adaptabilidade. Para esses autores, a resiliência de um sistema de saúde se caracteriza pela capacidade de antecipar e responder a crises. Nesse *framework*, a resiliência se manifesta de diversas formas: a consciência dos próprios recursos e das potenciais ameaças permite o planejamento estratégico; a integração com outros setores, como transporte e educação, amplia a capacidade de resposta; a diversidade de abordagens garante flexibilidade para enfrentar diferentes desafios; a autorregulação permite identificar e minimizar as interrupções dos serviços essenciais; e a adaptabilidade possibilita a transformação do sistema para melhor atender às novas demandas ^3^.

Utilizando dados quantitativos de bancos de dados públicos e dados qualitativos de relatórios técnicos de autoridades sanitárias brasileiras, Jatobá et al. ^13^ desenvolveram seu *framework*, baseando-se na lógica *Fuzzy* - um modelo matemático para determinar a pontuação em quatro capacidades de resiliência (monitoramento, antecipação, aprendizagem e resposta), além de um coeficiente agregado do potencial de resiliência na atenção à saúde.

Independentemente da estrutura proposta nos diferentes *frameworks*, a pesquisa sobre resiliência fornece uma perspectiva teórica para a compreensão dos sistemas de saúde como sistemas adaptativos complexos e destaca como as práticas de saúde precisam enfrentar, responder e se adaptar ao estresse, desafios ou demandas de acordo com suas capacidades ^13^. Ademais, a literatura relata a existência de fatores facilitadores e dificultadores ^14^ ^,^ ^15^ ^,^ ^16^ ^,^ ^17^ da resiliência de sistemas de saúde, que precisam ser mais bem compreendidos ^17^ ^,^ ^18^ ^,^ ^19^.

Baseado no exposto, o presente estudo tem como objetivo elencar as estratégias favoráveis e as barreiras da resiliência de sistemas de saúde, indicando os mecanismos pelos quais estas facilitam ou dificultam o seu alcance.

## Método

Trata-se de uma revisão de escopo realizada em julho de 2023 nas plataformas PubMed e Biblioteca Virtual em Saúde (BVS) e nas bases de dados Web of Science e SciELO, seguindo o protocolo do Instituto Joanna Briggs (JBI) ^20^, que contempla cinco etapas: (1) identificação e justificativa da questão de pesquisa; (2) estratégias para identificação dos estudos relevantes; (3) critérios de elegibilidade e de exclusão; (4) seleção e avaliação inicial dos estudos; e (5) análise dos dados.

### Identificação e justificativa da questão de pesquisa

Adotou-se a estratégia que considera População, Conceito e Contexto do objeto a ser pesquisado, sob o acrônimo PCC. Partiu-se da elaboração da questão de pesquisa: “O que as evidências científicas apontam como estratégias favoráveis e barreiras para o alcance de um sistema de saúde resiliente?”.

A análise da resiliência busca compreender a capacidade de os sistemas de saúde se prepararem para absorver, adaptar, aprender, transformar e se recuperar de choques. Nesta análise, destacam-se elementos como: liderança, tomada de decisões, coordenação de ações, disponibilidade e mobilização de recursos, que são elementos-chave para a gestão do sistema de saúde ^21^.

Desse modo, conhecer as estratégias favoráveis e as barreiras à resiliência de sistemas de saúde pode contribuir para o debate sobre as ações e as políticas necessárias para o sistema de saúde se tornar mais resiliente, por meio de mudanças no financiamento, governança e alocação de recursos, força de trabalho, insumos materiais e tecnológicos e, sobretudo, o modo de prestação de serviços de saúde.

### Estratégias para identificação dos estudos relevantes

A segunda etapa consistiu na escolha das bases de dados e elaboração das estratégias de busca. É importante ressaltar que cada base ou plataforma de dados tem suas peculiaridades, portanto, fazem-se necessárias adaptações nas estratégias de busca entre as diferentes bases ou plataformas, no intuito de se obter êxito na pesquisa. Desse modo, na plataforma PubMed empregaram-se os seguintes descritores e operadores booleanos: *(Resilience OR Resilient) AND (“Health System” OR “Brazilian National Health System”)*. Já nas bases Web of Science e SciELO, bem como no portal regional da BVS, utilizaram-se os descritores *Resilience* e *“Health System”*, intercalados pelo operador *booleano AND*.

### Critérios de elegibilidade e de exclusão

Foram considerados os estudos com informações, reflexões e discussões a respeito das características, conceitos, elementos, atributos, estratégias e/ou barreiras para o alcance de um sistema de saúde resiliente (conceito), do Brasil ou de outro país (contexto), envolvendo profissionais de saúde, usuários ou gestores (população). Foram selecionadas apenas as publicações no idioma português ou inglês e disponíveis na íntegra. O software de gestão de revisão sistemática Rayyan (https://www.rayyan.ai/) foi utilizado para viabilizar a seleção e análise dos estudos recuperados nas bases de dados.

Para extração dos dados a serem incluídos nesta revisão, exportou-se a lista de referências originada no Rayyan para uma planilha do programa Microsoft Excel (https://products.office.com/), que foi preenchida por duas revisoras de modo independente, em conformidade com o formulário específico para essa etapa, visando atender aos objetivos e à questão de pesquisa. As duas pesquisadoras são docentes de cursos da área da Saúde (Enfermagem e Odontologia) de universidades públicas (estadual e federal, respectivamente), com pós-graduação e atuação na área de Saúde Pública. No caso de divergências na seleção das publicações, havia uma terceira revisora (docente e pesquisadora em Saúde Pública em instituição federal de pesquisa).

### Seleção e avaliação inicial dos estudos

Como critérios de inclusão, foram considerados: pesquisas originais, ensaios teóricos, revisões de literatura, de escopo e sistemáticas, dissertações e teses, disponíveis na íntegra, sem limite temporal. Optou-se por incluir estudos de revisão com fins de ampliar a captação de publicações, haja vista a utilização de diferentes bases de dados nos diferentes estudos, que pode ampliar a inclusão de publicações sobre a temática. Aplicando-se os critérios de exclusão relativos ao tipo de documento, foram desconsiderados editoriais, resenhas, cartas, documentos governamentais, trabalhos de conclusão de curso de graduação e especialização e artigos não disponíveis on-line na íntegra.

Realizou-se a avaliação dos títulos e dos resumos de todos os estudos identificados, removendo-se os documentos não aderentes à revisão, usando-se o software Rayyan. Na primeira etapa, a triagem foi quanto à relevância do título e do resumo. Em seguida, os textos completos foram analisados quanto ao cumprimento dos critérios de inclusão e exclusão. Sendo assim, foram selecionados para leitura na íntegra as publicações cujos resumos elencavam ou discutiam aspectos relacionados a estratégias favoráveis e/ou barreiras para o alcance de um sistema de saúde resiliente. Publicações que mencionavam ou discutiam a respeito de pelo menos uma estratégia favorável ou barreira à resiliência de sistemas de saúde foram selecionadas. Dessa forma, minimizavam-se os vieses de seleção.

Elaborou-se um fluxograma sobre o processo de busca e seleção dos estudos desta revisão, com os resultados quantitativos de cada base de dados, estudos incluídos/excluídos e o total dos selecionados para avaliação e síntese (Figura 1). Essa etapa foi desenvolvida com base nas recomendações do guia internacional PRISMA para Revisões de Escopo (PRISMA-ScR) ^22^: agrupamento, síntese e apresentação dos dados.

Após a compilação das informações, a etapa seguinte consistiu na apresentação da síntese das evidências e dos resultados no formato de quadros.

**Figura 1:** Diagrama PRISMA de seleção de estudos.

Fonte: elaboração própria, a partir das recomendações do guia internacional PRISMA para Revisões de Escopo (PRISMA-ScR) ^22^.

### Análise dos dados

A análise dos dados se deu tomando como base as características explicitadas nos seis blocos de construção de sistemas de saúde resilientes preconizados pela OMS ^12^. O protocolo desta revisão está registrado na plataforma Open Science Framework (https://osf.io/jrkhb/files/osfstorage).

## Resultados

Um total de 1.533 artigos foram selecionados, sendo: 730 na plataforma PubMed, 612 na plataforma BVS, 163 na base de dados Web of Science e 28 na base SciELO. Como não se utilizou filtro temporal, foram encontradas publicações desde meados da década de 1990 até 2023. Os documentos foram avaliados, sendo excluídos os duplicados (438), o que resultou em 1.095 publicações. Após a leitura dos títulos e resumos, excluíram-se 868 por não discorrerem sobre a resiliência de sistemas de saúde no contexto do objeto da pesquisa. Das 227 publicações elegíveis para serem lidas na íntegra, 192 foram eliminadas por não atenderem à questão de pesquisa, ou seja, não fazerem menção ou discutirem acerca das estratégias facilitadoras e/ou entraves à resiliência de sistemas de saúde. Restaram, pois, 35 para compor a presente revisão de escopo. No Quadro 1 estão descritos autoria, ano de publicação, título e tipo/abordagem do estudo.

**Quadro 1:** Publicações incluídas na revisão de escopo - 2023 (n = 35).

ESTUDO (ANO)	TÍTULO	ABORDAGEM DO ESTUDO
Kruk & Freedman ^33^ (2008)	*Assessing Health System Performance in Developing Countries: A Review of the Literature*	Revisão sistemática da literatura
Kruk et al. ^4^ (2015)	*What Is a Resilient Health System? Lessons from Ebola*	Ensaio teórico
Ammar et al. ^30^ (2016)	*Health System Resilience: Lebanon and the Syrian Refugee Crisis*	Estudo de caso (revisão de literatura e análise quantitativa de dados secundários)
Gilson et al. ^32^ (2017)	*Everyday Resilience in District Health Systems: Emerging Insights from the Front Lines in Kenya and South Africa*	Estudo de caso de abordagem qualitativa (pesquisa documental, entrevistas em profundidade, grupos focais)
Abimbola et al. ^34^ (2019)	*The Impacts of Decentralization on Health System Equity, Efficiency and Resilience: A Realist Synthesis of the Evidence*	Revisão de literatura
Fridell et al. ^42^ (2020)	*Health System Resilience: What Are We Talking About? A Scoping Review Mapping Characteristics and Keywords*	Revisão de escopo
Gilson et al. ^43^ (2020)	*Organizational Change and Everyday Health System Resilience: Lessons from Cape Town, South Africa*	Estudo de caso de abordagem qualitativa (observação, entrevistas e análise qualitativa de dados secundários)
El Bcheraoui et al. ^31^ (2020)	*Assessing COVID-19 Through the Lens of Health Systems’ Preparedness: Time for a Change*	Ensaio teórico
Jamal et al. ^57^ (2020)	*Health System Resilience in the Face of Crisis: Analysing the Challenges, Strategies and Capacities for UNRWA in Syria*	Estudo de caso de abordagem qualitativa (entrevistas, grupos focais e análise documental)
Thomas et al. ^38^ (2020)	*Strengthening Health Systems Resilience: Key Concepts and Strategies*	Estudo teórico
Tumusiime et al. ^39^ (2020)	*Building Health System Resilience in the Context of Primary Health Care Revitalization for Attainment of UHC: Proceedings from the Fifth Health Sector Directors’ Policy and Planning Meeting for the WHO African Region*	Síntese de discussões de um fórum de planejamento e política de países africanos acerca de sistemas resilientes
Grimm et al. ^40^ (2021)	*Enhancing the Understanding of Resilience in Health Systems of Low- and Middle-Income Countries: A Qualitative Evidence Synthesis*	Revisão de literatura
Haldane et al. ^7^ (2021)	*Health Systems Resilience in Managing the COVID-19 Pandemic: Lessons from 28 Countries*	Revisão da literatura, documentos governamentais e entrevistas com especialistas
Sundararaman et al. ^41^ (2021)	*Pandemic Resilience and Health Systems Preparedness: Lessons from COVID-19 for the Twenty-First Century*	Ensaio teórico
Fleming et al. ^46^ (2022)	*Metrics and Indicators Used to Assess Health System Resilience in Response to Shocks to Health Systems in High Income Countries: A Systematic Review*	Revisão sistemática
Saulnier et al. ^47^ (2022)	*‘We Have A Plan For That’: A Qualitative Study of Health System Resilience Through the Perspective of Health Workers Managing Antenatal and Childbirth Services During Floods in Cambodia*	Estudo de abordagem qualitativa (entrevistas semiestruturadas)
Duby et al. ^48^ (2022)	*Adaptation and Resilience: Lessons Learned from Implementing a Combination Health and Education Intervention for Adolescent Girls and Young Women in South Africa During the COVID-19 Pandemic*	Estudo de abordagem qualitativa (intervenção e entrevistas em profundidade)
Mustafa et al. ^16^ (2022)	*COVID-19 Preparedness and Response Plans from 106 Countries: A Review from a Health Systems Resilience Perspective*	Pesquisa documental
Forsgren et al. ^15^ (2022)	*Health Systems Resilience in Practice: A Scoping Review to Identify Strategies for Building Resilience*	Revisão de escopo
Burau et al. ^14^ (2022)	*Health System Resilience and Health Workforce Capacities: Comparing Health System Responses During the COVID‐19 Pandemic in Six European Countries*	Estudo de caso de abordagem qualitativa (fontes secundárias e primárias e informações de especialistas)
Gooding et al. ^18^ (2022)	*How Can We Strengthen Partnership and Coordination for Health System Emergency Preparedness and Response? Findings from a Synthesis of Experience Across Countries Facing Shocks*	Pesquisa documental
Amiri et al. ^49^ (2022)	*Health System Resilience in the Eastern Mediterranean Region: Perspective on the Recent Lessons Learned*	Mesa redonda
Foroughi et al. ^52^ (2022)	*Hospitals During Economic Crisis: A Systematic Review Based on Resilience System Capacities Framework*	Revisão sistemática
Rawat et al. ^45^ (2022)	*The Contribution of Community Health Systems to Resilience: Case Study of the Response to the Drought in Ethiopia*	Estudo de caso de abordagem qualitativa (entrevistas com informantes-chave e discussões em grupos focais)
Cannedy et al. ^58^ (2022)	*Health System Resiliency and the COVID-19 Pandemic: A Case Study of a New Nationwide Contingency Staffing Program*	Abordagem qualitativa (entrevistas com diretores e líderes)
Paschoalotto et al. ^10^ (2023)	*Health Systems Resilience: Is It Time to Revisit Resilience After COVID-19?*	Estudo de abordagem qualitativa (entrevistas semiestruturadas com especialistas em sistemas de saúde)
Sagan et al. ^50^ (2023)	*Assessing Resilience of a Health System Is Difficult But Necessary to Prepare for the Next Crisis*	Ensaio teórico
Accoe et al. ^59^ (2023)	*Conditions for Health System Resilience in the Response to the COVID-19 Pandemic in Mauritania*	Estudo de caso de abordagem qualitativa (entrevistas semiestruturadas com informantes-chave das equipes de gestão)
Karreinen et al. ^60^ (2023)	*Living Through Uncertainty: A Qualitative Study on Leadership and Resilience in Primary Healthcare During COVID-19*	Estudo de caso de abordagem qualitativa (entrevistas semiestruturadas com líderes de sistemas de saúde)
Ako-Egbe et al. ^56^ (2023)	*Liberia Health System’s Journey to Long-Term Recovery and Resilience Post-Ebola: A Case Study of an Exemplary Multi-Year Collaboration*	Estudo de caso (pesquisa documental)
Mghamba et al. ^61^ (2023)	*The Use of Innovative Approaches to Strengthen Health System Resilience During the COVID-19 Pandemic: Case Studies from Selected Commonwealth Countries*	Estudo de caso de abordagem qualitativa (entrevistas com epidemiologistas e tomadores de decisão e pesquisa documental)
Hellevik et al. ^62^ (2023)	*Multisectoral Action Towards Sustainable Development Goal 3.d and Building Health Systems Resilience During and Beyond COVID-19: Findings from an INTOSAI Development Initiative and World Health Organization Collaboration*	Estudo de abordagem qualitativa (entrevistas com equipes de auditoria da OMS)
Lee et al. ^54^ (2023)	*Public Health Emergency Preparedness for Infectious Disease Emergencies: A Scoping Review of Recent Evidence*	Revisão de escopo
Ezzati et al. ^17^ (2023)	*Resiliency of the Iranian Healthcare Facilities Against the COVID-19 Pandemic: Challenges and Solutions*	Estudo de abordagem qualitativa (entrevistas semiestruturadas com 59 formuladores de políticas de saúde, gerentes e funcionários, e membros do corpo docente de uma Faculdade de Medicina)
Carvalho et al. ^19^ (2023)	*Transformative Dimensions of Resilience and Brittleness During Health Systems’ Collapse: A Case Study in Brazil Using the Functional Resonance Analysis Method*	Estudo transversal (análise de dados secundários por meio do método da Análise da Ressonância Funcional)

OMS: Organização Mundial da Saúde.

Tomando-se como base os seis blocos de construção de sistemas de saúde resilientes propostos pela OMS, a presente revisão de escopo possibilitou a identificação de alguns fatores considerados como facilitadores (estratégias) ou dificultadores (barreiras) para o alcance da resiliência. Para cada elemento facilitador ou dificultador elencado (descrito como “ideia central” nos quadros de resultados), foram também informados os mecanismos pelos quais estes facilitam ou dificultam a resiliência do sistema.

Com relação às estratégias favoráveis, verificam-se sete diferentes fatores, sendo quatro deles correspondentes aos “blocos de construção” de sistemas de saúde: força de trabalho, governança, sistemas de informação e financiamento. Paralelamente, descentralização, parcerias multissetoriais e comunidade envolvida surgem como fatores promotores da resiliência (Quadro 2).

**Quadro 2:** Estratégias favoráveis ao alcance da resiliência de sistemas de saúde.

IDEIA CENTRAL	SÍNTESE DE EVIDÊNCIAS DA LITERATURA	MECANISMOS DE CONTRIBUIÇÃO DA ESTRATÉGIA PARA O ALCANCE DA RESILIÊNCIA
1. Descentralização	A descentralização da tomada de decisão é uma importante capacidade organizacional de resiliência ^15^. Os governos descentralizados exibem a flexibilidade necessária e as respostas oportunas exigidas no gerenciamento de crises ^7^	Os múltiplos centros de governança em sistemas descentralizados permitem um efeito “absorvedor de choque”, de modo que as fraquezas de um podem ser compensadas por outros centros de governança ^34^. Os sistemas apresentaram maior flexibilidade quando as decisões foram tomadas mais próximas dos níveis operacionais do sistema de saúde ^15^
2. Força de trabalho comprometida e motivada	A resiliência do sistema de saúde melhora com a capacidade da força de trabalho em saúde e fortalecimento da integração das profissões de saúde na governança ^14^. Uma força de trabalho comprometida e responsiva é uma pré-condição para a resiliência ^30^ Mesmo com recursos escassos, a competência da força de trabalho em saúde deve ser suficientemente alta para manter as funções diárias do sistema ^42^ Uma força de trabalho comprometida, de tamanho adequado, flexível, motivada e treinada é crucial para criar e manter a resiliência ^15^ ^,^ ^17^ ^,^ ^38^ ^,^ ^41^ ^,^ ^59^ ^,^ ^60^	A força de trabalho é capaz de absorver as crescentes necessidades de saúde geradas pelos choques, adaptar-se para prestar serviços com recursos menores que o habitual e transformar-se de acordo com as mudanças no ambiente ^45^ Desenvolver o conhecimento e as habilidades dos indivíduos e de todo o sistema para lidar com a condição adversa por meio de programas de treinamento, prática e experiência leva à melhoria da resiliência do sistema ^58^ Uma forma de garantir uma força de trabalho adequada foi a realocação de pessoal, realizada através das fronteiras setoriais ^61^ Capacidade para rápida mobilização e redistribuição de profissionais de saúde e iniciativas para sustentar a produtividade dos profissionais de saúde, mesmo sob estresse, são importantes mecanismos para fortalecer a resiliência ^40^
3. Governança e liderança	Liderança e gestão são componentes essenciais e facilitadores da resiliência ^56^ ^,^ ^61^. A adaptação aos desafios emergentes requer uma liderança forte e flexível ^4^ Os líderes estão numa posição-chave na construção e manutenção da resiliência antes, durante e depois da crise ^61^. A liderança participativa é mais apropriada para organizações complexas, aumentando sua resiliência ^17^ ^,^ ^43^ As experiências destacam a importância da liderança para a resiliência cotidiana, exercida pelos gestores do sistema de saúde. Uma liderança eficaz deve ser capaz de demonstrar que o sistema de saúde é capaz de prevenir, detectar ou abordar eficazmente uma ameaça à saúde pública, sendo a sociedade o maior beneficiário ^43^	A liderança baseada em respeito pelos outros ou senso de dever, capacitando-os a serem decisivos e inovadores, gera um ambiente propício para os gerentes e profissionais de saúde da linha de frente, cuja confiança, comprometimento e motivação são essenciais em sistemas de saúde resilientes ^32^
4. Parcerias multissetoriais	O fortalecimento dos sistemas de saúde para resiliência precisa de uma abordagem integrada e colaboração multissetorial ^62^ Para uma resposta eficaz, são necessários esforços multissetoriais coordenados envolvendo atores públicos, privados e sociedade civil dentro e fora do setor de saúde ^16^ ^,^ ^54^ ^,^ ^63^ A importância da colaboração, comunicação e parceria multissetorial nacional e internacional é fundamental para construir e sustentar a resiliência dos sistemas de saúde ^63^. A boa governança baseada em uma parceria público-privada pode ser vista como uma pré-condição para a resiliência ^30^	A colaboração entre as partes interessadas, com envolvimento significativo entre o setor privado, a sociedade civil e os provedores de serviços, em parcerias multissetoriais formalizadas, pode ajudar a limitar as interrupções no acesso aos serviços de saúde e aumentar a resiliência do sistema a longo prazo ^52^ A boa governança, baseada em uma parceria público-privada, contribuiu para a garantir um fluxo constante de fundos ^32^
5. Comunidade envolvida	Só há resiliência dos sistemas de saúde com o envolvimento da comunidade que conhece os contextos locais em todos os domínios ^41^. Agentes comunitários de saúde podem aumentar a capacidade de um sistema para responder a situações de crise Os sistemas de saúde são preenchidos por profissionais de saúde e membros da comunidade; as relações e as colaborações entre eles influenciaram a resiliência ^49^ ^,^ ^60^	O envolvimento das comunidades aumenta a resiliência do sistema de saúde por meio do fornecimento de informações críticas, ideias e *feedback* para melhorar a capacidade do sistema de responder rapidamente às necessidades das pessoas durante choques e tempos de calmaria ^14^ ^,^ ^58^ A transparência, a confiança do público, a comunicação aberta e a legitimidade do sistema de saúde são importantes para a resiliência. Se o sistema de saúde estabelecer relações adequadas e transparentes com as pessoas, elas serão mais cooperativas em tempos de crise ^17^
6. Sistema de informação/monitoramento e vigilância eficaz	Um sistema de vigilância digitalizado e robusto, perfeitamente integrado aos sistemas de informação de gestão de saúde é um elemento essencial para a construção de resiliência. É importante usar dados para prever, preparar e responder eficazmente às mudanças, tomar decisões baseadas em evidências, fornecer monitoramento e avaliação regulares e realizar análises de sistema ^17^ ^,^ ^46^ A tecnologia e a informação ganham relevância ao interagir com todas as dimensões da resiliência do sistema de saúde melhorando o seu desempenho ^10^ A informação de saúde em tempo real é inestimável não apenas para fornecer alertas precoces de um surto, mas também para rastrear a propagação da epidemia, prever e preparar o sistema de saúde ^33^. Mobilizar sistemas de monitoramento, detecção digital de saúde e expandir a capacidade de identificação precoce de ameaças é mandatório para um desempenho resiliente ^19^ Sistemas de vigilância precisam garantir que os dados cheguem a todas os interessados e sejam rapidamente transformados em informações para tomada de decisões ^31^	Um sistema eficaz de vigilância de doenças relata não apenas eventos súbitos de saúde incomuns, mas também dados de rotina sobre padrões de morbidade de todos os profissionais de saúde. Identifica áreas onde o acesso a outros serviços essenciais de saúde foi comprometido e precisam ser atendidos com urgência ^46^ Fluxos de informação diligente permite reações ágeis dos gestores na tomada de decisões ^39^
7. Financiamento (investimentos em recursos humanos, materiais e financeiros - sustentabilidade)	A resiliência necessita de recursos suficientes e disponíveis em caso de crise ^17^ ^,^ ^39^ Recursos humanos, financeiros, materiais e tecnológicos para enfrentamento de uma crise, são fatores capacitadores da resiliência do sistema ^58^ ^,^ ^64^	Em caso de crise, ter recursos suficientes e bem distribuídos economiza tempo para o desenvolvimento de capacidades e proporciona resiliência ^19^ Mecanismo para mobilizar rapidamente recursos através da realocação e/ou fundos de parceiros para responder a ameaças é uma intervenção que favorece sistemas resilientes ^40^ Importante desenvolver capacidade de fabricação doméstica de insumos e ecossistemas de inovação necessários para a segurança da saúde e a autossuficiência ^40^. A resiliência do sistema de saúde requer a promoção da sustentabilidade e investimentos para promover pesquisa e inovação ^62^

Em contraposição, cinco fatores são listados como entraves à resiliência de sistemas de saúde, três deles indicando exatamente o oposto de estratégias facilitadoras já descritas, relacionados aos “blocos de construção” preconizados pela OMS ^12^: liderança, força de trabalho e sistemas de informação ineficazes. Adicionalmente, surgem dois dificultadores: falta de solidariedade global e falha em aprender com as lições passadas. Cabe assinalar que, na presente revisão, as barreiras tiveram menor representatividade que as estratégias favoráveis à resiliência de sistemas de saúde (Quadro 3).

**Quadro 3:** Barreiras para o alcance da resiliência de sistemas de saúde.

IDEIA CENTRAL	SÍNTESE DE EVIDÊNCIAS DA LITERATURA	MECANISMOS DE AÇÃO DA BARREIRA PARA DIFICULTAR O ALCANCE DA RESILIÊNCIA
1. Liderança/Governança ineficaz	A má gestão e a liderança fraca, afetando o envolvimento de diferentes setores e níveis do governo, foram identificadas como dificultadoras da coordenação para resposta a estressores ^15^ ^,^ ^16^ Os desafios de resiliência mais importantes foram: sistema de gestão fragmentado ^60^, tomada de decisão centrada na pessoa, falta de coordenação, liderança ineficaz, falta de comprometimento dos gestores ^15^	A falta de diretrizes centrais dificulta o desempenho resiliente, pois a tomada de decisões e as políticas se tornam mais desgastantes ^17^ A falta de coordenação intrassetorial e intersetorial é um grande desafio para a liderança, uma vez que aumenta a probabilidade de atrasos e perda de tempo em projetos e programas. O egocentrismo e o partidarismo impedem os gestores de desenvolver uma visão nacional ^15^
2. Sistema de informação/monitoramento e avaliação ineficaz	Monitoramento e avaliação inadequados são importantes desafios de resiliência ^15^	A falta de dados/informações/comunicação e transparência governamental destes reduz a tomada de decisão eficaz e o capital social, sendo um desafio importante para a resiliência ^15^
3. Força de trabalho ineficaz	O processo ineficaz de seleção e recrutamento de funcionários, a falta de conscientização entre eles, sua baixa competência e habilidade, despreparo e falta de apoio são os principais desafios ^15^	Os funcionários são os recursos mais importantes de uma organização, que devem ser desenvolvidos e capacitados para se alcançar os objetivos organizacionais ^15^
4. Falta de solidariedade global	A diminuição da solidariedade global pode ter afetado a resiliência dos sistemas de saúde ^42^	A fragilidade dos sistemas de saúde ocorre ao mesmo tempo em que aumentam as incertezas, causadas por uma menor solidariedade global e por visões divergentes dos líderes mundiais ^42^
5. Falha em aprender com as experiências (lições) passadas (falta de cultura da resiliência)	Falha em aproveitar as experiências e lições aprendidas e falta de aprendizado organizacional são desafios para a resiliência dos sistemas de saúde ^15^	Se as organizações não compreenderem a resiliência, não conseguirão implementá-la, não compreenderão as suas definições e conceitos, sofrerão com a entropia, tornar-se-ão obsoletas ^15^

## Discussão

A despeito dos diversos significados conferidos ao termo resiliência de sistemas de saúde, tal terminologia foi tratada neste estudo como um atributo dos sistemas de saúde pública que permite que eles se adaptem, absorvam e respondam a demandas rotineiras e extraordinárias sem comprometer a prestação de serviços universais e equitativos ^23^, possibilitando, ainda, que eles se reorganizem, embasados pelas lições aprendidas durante crises ^4^. Considerando situações de crises, os sistemas de saúde pública devem ser mais adaptativos, proativos e responsivos para garantir cobertura universal, abrangente e equitativa de serviços essenciais e evitar o colapso ^24^.

Jatobá et al. ^23^ enfatizam que o debate sobre a resiliência dos sistemas de saúde persiste mesmo imerso em incertezas sobre seu significado, e sem o consenso sobre sua operacionalização em contextos de emergências em saúde pública, mas é fundamental que a resiliência não seja tratada apenas como um conceito reativo, mas também proativo, especialmente quando se refere à capacidade dos sistemas de saúde de responder a circunstâncias incomuns. Cabe também salientar que a resiliência não deve ser vista como resignação diante de condições adversas por meio de respostas adaptativas, mas como propulsora do desenvolvimento contínuo de capacidades de monitoramento, antecipação e aprendizado, que impulsionam a transformação constante de sistemas de saúde ^6^.

Majoritariamente, a literatura que trata de resiliência em saúde pública se concentra em eventos catastróficos, como pandemias e epidemias, ao invés da entrega contínua de serviços abrangentes de saúde ^25^. Na presente revisão, a noção de resiliência no funcionamento de sistemas de saúde também foi prioritariamente direcionada a emergências em saúde pública, possivelmente justificado pelo momento de crise sanitária vivenciado com a pandemia COVID-19.

Nesta revisão de escopo, emergiram na literatura pesquisada fatores considerados como estratégias favoráveis ao alcance da resiliência de sistemas de saúde, tais como a governança e o financiamento. De acordo com Haldane et al. ^7^, diversos países aumentaram sua resiliência por meio de governança, financiamento e mecanismos de responsabilização, ao implementar pacotes de apoio a grupos vulneráveis, protegendo e apoiando populações de baixa renda e, reduzindo, assim, a sobrecarga nos sistemas de saúde. Podem ser citados os programas de saúde específicos para povos indígenas em países como o Brasil ^26^ e o Canadá ^27^; políticas diferenciadas para populações migrantes e marginalizadas ^28^ e pessoas em situação de rua; e a expansão de programas de agentes comunitários de saúde em áreas vulneráveis no Reino Unido ^29^.

A boa governança, sobretudo aquela intersetorial, merece destaque ^30^, ainda que muitas vezes seja negligenciada no contexto de respostas a choques ^31^. Sistemas de saúde bem governados estão melhor preparados para enfrentar tanto desafios de saúde a longo prazo quanto emergências repentinas. Sendo o cerne na formulação de políticas, uma governança eficaz em saúde pública é fundamental para garantir uma alocação adequada de recursos e promover a colaboração intersetorial para o enfrentamento dos determinantes sociais da saúde, incluindo a priorização de fatores como vulnerabilidade social e diversidade ^6^.

A liderança participativa é mais apropriada para organizações adaptativas complexas e pode aumentar sua resiliência ^17^. Kruk et al. ^4^ ressaltam, ainda, a importância de uma liderança forte e flexível para o enfrentamento dos desafios emergentes, não somente em tempos de crise, mas também em tempos normais (resiliência cotidiana). Corroborando, Gilson et al. ^32^ destacam que uma liderança baseada na confiança, que tem como prioridade capacitar, treinar e motivar seus liderados, especialmente aqueles da linha de frente, torna-os decisivos e inovadores, contribuindo para aumentar a resiliência do sistema de saúde.

A descentralização da tomada de decisão, estratégia fortemente elencada nesta revisão de escopo, constitui-se importante capacidade organizacional de resiliência, já que propicia a flexibilidade necessária para as respostas exigidas no gerenciamento de crises ^15^ ^,^ ^33^. Sendo assim, organizações resilientes utilizam a descentralização, auto-organização e tomada de decisão compartilhada para permitir flexibilidade e adaptação ^30^. Abimbola et al. ^34^ destacam que os múltiplos centros de governança em sistemas descentralizados possibilitam o amortecimento do choque, de modo que as fraquezas de um centro de governança possam ser compensadas pela governança fortalecida de outros centros.

No caso brasileiro, explorar papéis, responsabilidades e atuação das instâncias de gestão entre esferas de governo diante de situações de crise é tema fundamental para agenda de pesquisa sobre a resiliência no Sistema Único de Saúde (SUS). Especialmente em situações de crise, é importante compreender como as esferas de gestão do SUS utilizam informações e evidências para tomada de decisão, como ocorre a participação de atores-chave do sistema e como se realiza a coordenação da resposta entre setores público e privado do sistema de saúde ^35^.

De acordo com Bloom et al. ^36^, resiliência na saúde pública é uma capacidade contextual, dependente em grande parte da construção de sistemas nacionais de saúde pública e universal. Isso requer esforços contínuos para fortalecer estruturas governamentais e promover parcerias intersetoriais. Desse modo, a resiliência é uma característica essencial da governança democrática que abrange a geração de habilidades e a resposta a ameaças e pressões políticas. Portanto, as respostas dos sistemas de saúde não podem ser isoladas das políticas públicas, das mudanças e do surgimento de rupturas e conflitos ^37^.

Na presente revisão de escopo, constata-se a colaboração multissetorial contínua como fator que contribui para suprir as lacunas persistentes em todos os níveis do sistema de saúde ^16^. Para uma resposta eficaz, são necessários esforços multissetoriais coordenados envolvendo atores públicos, privados e sociedade civil, dentro e fora do setor de saúde ^38^. Desse modo, parcerias multissetoriais formalizadas podem evitar interrupções no acesso aos serviços de saúde, aumentando, assim, a resiliência do sistema a longo prazo ^39^, especialmente em tempos de crise ^40^. Ademais, reconhecer que os sistemas de saúde estão inseridos em outras estruturas complexas (por exemplo, sistemas políticos, econômicos, judiciários, sociais e ecológicos) e em escalas diversas (níveis local, subnacional, nacional e internacional) facilita a compreensão de como os sistemas de saúde são afetados por fatores que, embora não pareçam, estão diretamente relacionados à saúde pública ^7^ ^,^ ^41^.

O alcance da resiliência como capacidade do sistema de saúde não é um processo simples, pois requer coordenação entre recursos materiais e humanos e alocação de tarefas de acordo com a natureza da ameaça. Atores específicos no sistema de saúde lidam com diferentes tipos de eventos, interagindo com pacientes, gestores públicos e privados, em interações que dependem de motivações e interesses das pessoas, bem como das estruturas dentro das quais os sistemas de saúde operam, de arranjos financeiros e políticos estáveis, abertos e responsivos à adaptação para melhorar o desempenho do sistema ^23^.

De acordo com Jatobá & Carvalho ^25^, o desenvolvimento contínuo de capacidades adaptativas, absorventes e preventivas permite que os profissionais de saúde lidem com situações desconhecidas de forma proativa, migrando de abordagens tradicionais para comportamentos mais resilientes. Contudo, sistemas que dependem apenas da resiliência dos atores, especialmente os que atuam na linha de frente, sobrecarregam os trabalhadores, levando à instabilidade e à perda do controle ^37^. É preciso que arranjos de alto nível (financeiros e políticos), estáveis, responsivos e adaptativos promovam, por meio do aprendizado, transformações que acarretem o bom desempenho do sistema ^23^.

Nesta revisão de escopo, uma força de trabalho comprometida/motivada é considerada importante elemento facilitador da resiliência, na medida em que contribui para a absorção das crescentes necessidades de saúde geradas pelos choques, adaptando-se para prestar serviços com recursos muitas vezes menores do que o habitual e transformando-se de acordo com as mudanças no ambiente gerado pelo choque ^34^. A flexibilização de tarefas e o trabalho em equipe também são fundamentais para o alcance da resiliência ^42^. Ademais, preparar os indivíduos para lidar com condições adversas por meio de programas de treinamento pode contribuir para a melhoria da resiliência do sistema ^43^.

A governança centrada na resiliência em saúde pública enfatiza a inclusão da participação social nos processos de formulação de políticas, garantindo que aqueles afetados pelos determinantes sociais também participem da elaboração das políticas e programas que impactam suas vidas ^6^. O desempenho resiliente depende de políticas públicas que se baseiam em uma compreensão abrangente das realidades da comunidade ^35^ ^,^ ^44^. Portanto, uma adequada governança é essencial para abordar os fatores estruturais que influenciam os desfechos em saúde, especialmente para populações desfavorecidas. Ao focar na equidade e nos resultados em saúde, a governança pode impulsionar mudanças sistêmicas que ajudam a reduzir disparidades em saúde ^6^.

No presente estudo, o envolvimento da comunidade surge como destacada estratégia para o alcance da resiliência de sistemas de saúde, conforme enfatizado por Rawat et al. ^45^, segundo os quais comunidades efetivamente engajadas, proativas e vinculadas aos seus sistemas de saúde são essenciais para a construção de resiliência e para uma resposta bem-sucedida. Ressalta-se nesse contexto a confiança e o sentimento de pertencimento da comunidade, que podem ser construídos mediante seu envolvimento nos processos de formulação de políticas e na gestão de serviços de saúde ^46^ ^,^ ^47^.

Com relação aos sistemas de informação, estratégia abordada por vários autores nesta revisão de escopo ^17^ ^,^ ^48^ ^,^ ^49^ e enfatizada por Kruk & Freedman ^33^, estes não apenas fornecem alertas precoces de um surto, mas também podem rastrear a propagação de epidemias, prevendo e preparando o sistema de saúde para o surto ^50^. Fukuma et al. ^51^ chamam atenção para o fato que, durante o grande terremoto de Fukushima (Japão), avanços na tecnologia de comunicação de informação (TIC) desempenharam papel fundamental na comunidade local como fonte alternativa de informação e plataforma de comunicação. Faz-se necessário, entretanto, o estabelecimento de um fluxo claro de informação para evitar o risco de decisões erradas, registros incompletos ou duplicidade e garantir que a informação seja compartilhada de forma adequada e em tempo hábil para permitir uma resposta rápida e correta ^52^. No tocante à COVID-19, Carvalho et al. ^19^ destacam que essa pandemia expôs a fragilidade de sistemas de saúde nos níveis nacional e subnacional. Portanto, mobilizar sistemas de monitoramento e expandir a capacidade de identificação precoce de ameaças transfronteiriças tornou-se mandatório para um desempenho resiliente do sistema de saúde ^52^.

De acordo com Jatobá ^6^, a capacidade institucional por si só não é suficiente para alcançar o desempenho resiliente em saúde pública. Experiências recentes mostraram que uma grande capacidade institucional não garante um comportamento resiliente ^25^ ^,^ ^53^. Durante a pandemia de COVID-19, alguns países desenvolvidos com grande capacidade institucional tiveram dificuldades para controlar a rápida disseminação do vírus SARS-CoV-2. A resiliência depende de como os sistemas de saúde atuam sob adversidades, gerenciando suas capacidades institucionais em prol da continuidade das funções essenciais de saúde pública ^3^ ^,^ ^44^. Isso foi evidente em países como Estados Unidos e Brasil durante a pandemia, que, apesar de possuírem recursos, enfrentaram dificuldades operacionais devido a estratégias conflitantes em níveis decisórios superiores ^19^.

Na análise do caso brasileiro, Massuda et al. ^21^ apontam as políticas de austeridade como elementos que fragilizaram a resposta do SUS à pandemia, não obstante a ampla e abrangente estrutura construída ao longo dos anos. Os autores destacam o quadro conjuntural decorrente do desastroso papel do governo, mas também problemas crônicos do SUS, como o subfinanciamento, entre outros.

No presente estudo, investimentos em recursos materiais e humanos emergiram como fatores promotores da construção de sistemas de saúde resilientes, mitigando os impactos de uma emergência de saúde e reduzindo a interrupção dos serviços essenciais de saúde pública ^54^. A literatura aponta, entretanto, a necessidade de investimentos na capacidade de fabricação doméstica e na construção dos ecossistemas de inovação necessários para a segurança da saúde e a autossuficiência ^50^.

Corroborando esse debate, Nyasulu et al. ^55^ apontam a infraestrutura de sistemas de saúde dos países como determinante para a resposta a pandemias, independentemente dos recursos disponíveis. Os autores citam o exemplo do Malawi: embora possuísse um sistema de saúde desprovido de recursos, as estruturas de saúde comunitárias existentes no país contribuíram para a resiliência dos sistemas de saúde durante a pandemia COVID-19.

Contudo, como destacam Paschoalotto et al. ^35^ para o contexto brasileiro, diante de uma situação de crise é importante explorar como ocorre a destinação de recursos financeiros públicos e privados e como as desigualdades de financiamento influenciam a resiliência do SUS, bem como o fluxo de recursos financeiros entre as esferas federativas na resposta à situação de crise e os critérios utilizados para alocação de recursos.

No tocante às barreiras impostas à resiliência de sistemas de saúde, a baixa competência e habilidade dos trabalhadores aliada à falta de apoio a essa categoria foram os principais desafios elencados por Ezzati et al. ^17^. Os autores ressaltam, ainda, a importância de uma “cultura da resiliência”, que valoriza o treinamento e a mudança de comportamento em prol da implementação da resiliência do sistema de saúde.

A gestão ineficaz e desprovida de uma boa liderança consistiu em um grande desafio para a liderança e a gestão durante a pandemia de COVID-19 ^17^. Abordagens fragmentadas e isoladas, por exemplo, dificultaram os esforços para tornar os sistemas de saúde mais resilientes ^56^. Ademais, a falta de solidariedade provocou fendas na conjuntura entre a saúde global e a nacional e disparidades entre a resposta local e global dos países no combate à pandemia ^31^. Por fim, não aproveitar as experiências e lições aprendidas destacou-se como uma barreira para a resiliência de sistemas de saúde ^17^.

Fridell et al. ^42^ apontam que a maioria dos estudos sobre resiliência se concentra na absorção de choques sistêmicos em prazo relativamente curto, em vez de aprendizado e transformação do sistema a longo prazo. Para reverter esse quadro, maior atenção deve ser dada a fatores que favoreçam a capacidade do sistema de aprender com choques, para que possa, no futuro, responder de modo eficaz ^39^.

A Guiné é exemplo de país que utilizou sua experiência com base nas lições aprendidas para conter os impactos negativos da COVID-19. Após o choque causado pela epidemia de Ebola (2014-2016), um plano foi colocado em prática para treinar epidemiologistas locais. De acordo com Tumusiime et al. ^39^, não houve relatos de desafios ou dificuldades relativos à infraestrutura, equipamentos e outros recursos. Evidentemente, possuir essas estruturas antes da COVID-19 proporcionou vários benefícios, como a redução de despesas. No entanto, com a COVID-19 lições adicionais foram aprendidas, como a consciência da necessidade de se ter disponíveis dados de mortalidade e morbidade em tempo real ^39^.

De acordo com Jatobá et al. ^13^, o alcance de metas de saúde ambiciosas requer sistemas de saúde fortes, funcionais e inclusivos. Os blocos de construção de sistemas de saúde resilientes propostos pela OMS destacam apenas os elementos essenciais, sendo necessários outros esforços e investimentos em prol do acesso e da cobertura universais. Entretanto, estes constituem um ponto de partida útil para gestores e autoridades sanitárias.

Não obstante a relevância do tema desta revisão de escopo, que seguiu *pari passu* o protocolo inicialmente proposto, o estudo apresenta algumas limitações, como ter restringido os idiomas ao português e inglês e não ter utilizado a totalidade de bases/plataformas de dados disponíveis, tampouco a literatura cinzenta. Ademais, pode-se listar como um dificultador a extensa variabilidade no conceito do termo resiliência, aliado à forte tendência de utilizá-lo no contexto de crises, haja vista a recente pandemia de COVID-19, fazendo com que o pico das publicações se concentrasse nos anos de 2020 a 2022.

A despeito desses aspectos, a pesquisa atingiu seu objetivo, possibilitando uma compilação criteriosa de publicações sobre a temática, contribuindo, assim, para a compreensão das estratégias e das barreiras para o alcance da resiliência de sistemas de saúde, tão necessária para o enfrentamento de futuros choques/estressores na área da Saúde.

## Considerações finais

Descentralização do sistema, força de trabalho comprometida e motivada, governança e liderança eficazes, parcerias multissetoriais, comunidade envolvida, sistema de informação adequado e investimentos que favoreçam a sustentabilidade do sistema de saúde são estratégias que contribuem para o alcance da resiliência. Por outro lado, gestão ineficaz/falta de liderança, monitoramento inadequado, força de trabalho ineficaz, falta de solidariedade global e falha em aprender com as experiências passadas constituem barreiras ou entraves à resiliência de sistemas de saúde.

Entender os componentes de um sistema de saúde resiliente e como construí-los, mantendo o fornecimento dos serviços essenciais, é imprescindível para a preparação de sistemas de saúde diante das intempéries. Assim, a compreensão das estratégias e barreiras pelo conjunto de atores é fundamental para a construção de um sistema de saúde mais resiliente e resolutivo, capaz de enfrentar estressores crônicos e agudos, possibilitando que a população possa usufruir de serviços de saúde em crescente qualificação.
